# Associations of Low‐Level Prenatal Alcohol and Cannabis Exposure With Adolescent Cognitive Trajectories

**DOI:** 10.1111/acer.70297

**Published:** 2026-04-07

**Authors:** Katie J. Paige, Nolan E. Ramer, Emma K. Devine, Omid Kardan, Louise Mewton, Lindsay M. Squeglia, Mary M. Heitzeg, Alexander S. Weigard

**Affiliations:** ^1^ Psychiatry University of Michigan Ann Arbor Michigan USA; ^2^ Department of Psychiatry and Behavioral Sciences, College of Medicine Medical University of South Carolina Charleston South Carolina USA; ^3^ The Matilda Centre for Research in Mental Health and Substance Use University of Sydney Sydney New South Wales Australia

**Keywords:** adolescence, adolescent cognitive abilities, cognitive outcomes, prenatal alcohol exposure, prenatal cannabis exposure

## Abstract

**Background:**

No studies have examined the differential and combined effects of prenatal alcohol and cannabis exposure (PAE/PCE) on longitudinal trajectories of adolescent cognitive development. Further, previous alcohol research is mixed, with some evidence for negative PAE effects on cognition and other studies reporting null or positive associations. This study examined associations between PAE, PCE, and growth trajectories of adolescent cognition in a large, diverse sample.

**Methods:**

*N* = 11,029 adolescents from the Adolescent Brain Cognitive Development℠ Study completed the NIH Toolbox cognitive battery at baseline (*M*_age_ = 9.95), two‐year follow‐up (*M*_age_ = 11.95), and four‐year follow‐up (*M*_age_ = 14.07). Retrospective parent report of PAE and PCE was assessed at baseline. Univariate growth trajectories were estimated for cognitive measures: Pattern Comparison, Picture Sequence Memory, Oral Reading, Flanker Task, and Picture Vocabulary. Cross‐product terms for PAE and PCE tested combined use.

**Results:**

Most mothers reported no prenatal alcohol (*n* = 8257; 74%) or cannabis (*n* = 10,812; 94%) use. Overall, use was low: across pregnancy, women reporting any alcohol use averaged 33.31 drinks, and those reporting any cannabis use averaged 33.00 use occasions. Before including covariates, there were negative main effects of PCE and positive main effects of PAE on intercepts for all five cognitive domains. There was little evidence for PCE/PAE effects on slopes for cognition. After adding covariates, no negative effects of PCE remained. Small positive PAE effects on intercepts for multiple domains persisted. Cross‐product terms for combined exposure were not significant.

**Conclusions:**

Little evidence emerged for negative effects of low PAE, PCE, or combined exposure on adolescents' cognitive development after accounting for sociodemographic factors. Light drinking in families with social features positively associated with cognitive ability may result in few negative consequences. This study is the first to demonstrate weak evidence for adverse differential and combined low‐level PAE and PCE effects on the development of adolescent cognition.

## Graphical abstract

Little evidence emerged for negative effects of low level prenatal alcohol, cannabis, or combined exposure on adolescents' cognitive development after accounting for sociodemographic factors. Families with social features positively associated with cognitive ability (e.g., education) may engage in low level drinking during pregnancy with few negative consequences on offspring cognition.

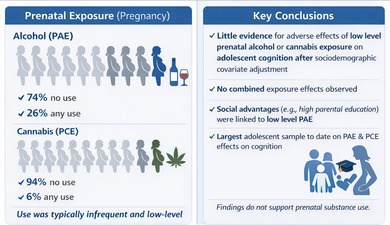

## 1 Introduction

Adolescence is a critical period in which environmental influences shape the development of cognitive abilities that are essential for adaptive functioning throughout an individual's lifespan (Galván 2021; Paige et al. 2025; Paige and Colder 2020). The potential long‐term impacts of prenatal alcohol and cannabis exposure (PAE/PCE) on adolescent cognitive abilities are of significant public health interest (English and Greyson 2022; Luciana et al. 2018), especially due to the common global prevalence (10%) of PAE (Popova et al. 2017) and increasing prevalence of PCE in the United States (U.S.) (Ko et al. 2015; Young‐Wolff et al. 2022). It is well‐known that heavy drinking during pregnancy is a risk factor for fetal alcohol spectrum disorder, which is characterized by cognitive impairments (Kodituwakku 2009). With respect to the mechanism by which PAE may have causal effects on cognitive development, research has demonstrated that alcohol has the ability to pass through the placental and blood–brain barriers to access the fetal brain and activate GABA receptors (Olney et al. 2002), which may induce long‐term changes in offspring brain and cognitive function. Further, preclinical and human studies have suggested that PAE may cause cognitive impairment through a variety of mechanisms, including the disruption of synaptic plasticity, long‐term potentiation, neurogenesis, and mitochondrial dysfunction (*for a review*, see Alhowail 2022). However, it should be noted that this research has been primarily conducted in heavy drinking samples, and the links between low levels of PAE and offspring cognitive development are less clear (Flak et al. 2014; Testa 2003).

Similarly, the main psychoactive constituent of cannabis, Δ9‐tetrahydrocannabinol (THC), is able to cross the placenta and enter the fetus (Hutchings et al. 1989; Maia et al. 2020). Thus, it is thought that cannabis may impact offspring cognitive development by acting on fetal endocannabinoid systems. Specifically, endocannabinoids and their CB1 receptors play a critical role in early brain development, guiding processes such as neural progenitor proliferation, migration, and synaptogenesis. Exposure to THC during pregnancy may disrupt these pathways, as CB1 receptors are present in the fetal brain during the first trimester, particularly in regions important for cognition such as the hippocampus and prefrontal cortex (Berghuis et al. 2007; Wang et al. 2003). Indeed, preclinical studies show that prenatal THC exposure alters dopamine and glutamate systems, which are both essential for cognitive function, resulting in long‐term changes associated with cognitive impairments (Campolongo et al. 2007; Suárez et al. 2004). In summary, these findings suggest that PCE may disrupt multiple neurodevelopmental pathways with enduring effects on cognitive outcomes. However, several critical questions regarding the differential and combined effects of PAE and PCE on adolescent cognitive development remain unanswered.

First, prior research on potential effects of PAE/PCE on youths' cognition has been concentrated in infancy and childhood (Jacobson et al. 2021; Sorkhou et al. 2024; Thompson et al. 2023), and adolescent‐focused studies have only examined associations between PAE and/or PCE and cognition at one or two timepoints (Lees et al. 2020; Thompson et al. 2023; Torres et al. 2020). Therefore, the impact of PAE/PCE on *changes* (growth trajectories) in adolescent cognition across time is unknown. This is a critical gap, as substance‐induced deficits may emerge as offspring age (Hagan et al. 2016; Kully‐Martens et al. 2012). Adolescence is a period characterized by qualitative neurodevelopmental changes, during which several brain systems that support cognitive abilities undergo substantial reorganization (Ordaz et al. 2013; Paige et al. 2024). Indeed, Dual Systems Models emphasize the protracted maturation of the cognitive control system, supported by prefrontal cortical networks, which show marked functional integration during adolescence (Casey et al. 2008; Steinberg 2008). Therefore, neurobiological disruptions occurring earlier in development may not manifest behaviorally until these systems reach a sufficient level of maturity. As previously discussed, prenatal exposure to alcohol and/or cannabis may alter fundamental neurodevelopmental processes, including synaptic plasticity, neurogenesis, mitochondrial function, and the development of dopamine and glutamate signaling systems. It is possible that disruptions to these processes may remain latent in childhood but become increasingly evident during adolescence, when higher order cognitive systems dependent on these neural substrates are recruited more heavily. Moreover, the rapid neurocognitive development that occurs across the adolescent years suggests that youth exposed to substances in utero may continue to benefit from interventions to promote healthy cognition across this developmental period (Luna 2017). This is supported by some empirical evidence, as a recent Adolescent Brain Cognitive Development^℠^ Study (ABCD Study) using two assessments of cognition (*M*_ages_ = 9.91 and 12.00) concluded that the effects of PCE may become more magnified as development progresses (Hiraoka et al. 2023). In summary, longitudinal research on associations between PAE and PCE, and cognitive development that allows for more detailed modeling of growth (i.e., assesses cognition across at least three timepoints) across adolescence is needed.

Second, although detrimental effects of heavy PAE (i.e., binge‐drinking) on offspring cognition are well‐established (Flak et al. 2014; Jacobson et al. 2021), the most disabling being fetal alcohol spectrum disorder (FASD) (Kodituwakku 2009), it is less clear whether low‐level PAE adversely impacts cognition. In fact, a growing number of studies have reported small, *positive* effects of low‐level PAE on cognitive outcomes across meta‐analyses (Flak et al. 2014; Testa 2003) and ABCD (Gu et al. 2024; Lees et al. 2020). It is hypothesized that positive associations result from residual confounding from demographic variables; indeed, low‐level PAE is consistently associated with higher socioeconomic status (Flak et al. 2014; Lees et al. 2020; Shmulewitz and Hasin 2019; Testa 2003). Thus, offspring of women who endorse low‐level PAE likely benefit from a wide array of positive socio‐environmental influences (e.g., high‐quality schooling and nutrition) on cognitive development. Further research is therefore required to better understand the link between PAE on adolescent cognitive development in the context of potential confounders.

Specifically, while previous studies have accounted for the influence of these sociodemographic confounders through covariate adjustment in regression approaches (Gu et al. 2024; Jacobson et al. 2024), fewer have used more stringent statistical approaches, such as demographic matching analyses. Whereas covariate adjustment statistically adjusts for the influence of confounders in the analysis stage, demographic matching ensures groups (e.g., adolescents with PAE vs. adolescents with no PAE) are equivalent on key sociodemographic characteristic *before* analysis. This study addresses this gap by including a wide range of sociodemographic variables in main analyses testing study hypotheses, as well as performing sensitivity analyses using demographic matching. Thus, results may shed new insights into the role of the sociocultural context in which PAE occurs when considering associations with offspring adolescent cognitive development.

Similarly, questions regarding the effects of PCE on offspring outcomes remain unanswered (Sorkhou et al. 2024). Two systematic reviews found little evidence for PCE effects on cognitive outcomes across infancy to early adulthood (Thompson et al. 2023; Torres et al. 2020). However, a recent meta‐analysis revealed mixed findings (Sorkhou et al. 2024). While PCE did not adversely impact the majority of cognitive outcomes in infancy and early childhood after accounting for covariates, qualitative synthesis suggested that PCE was linked to poor attention. Notably, all aforementioned studies focused on the absence versus presence of PCE, with less discussion about differences in effects across varying frequencies of cannabis use during pregnancy.

Third, while cannabis use and co‐use of alcohol and cannabis during pregnancy is increasing (Mejia et al. 2024; Volkow et al. 2019; Young‐Wolff et al. 2022), no previous studies have tested combined effects of PAE and PCE on trajectories of adolescent cognition. This is notable because prenatal exposure to alcohol and cannabis are each independently associated with risks for adverse neurocognitive development; thus, it is reasonable to hypothesize that their combined (additive) effects may also be detrimental. There is emerging evidence from preclinical research that prenatal exposure to both alcohol (ethanol) and THC leads to unique impacts on development of the hippocampus (Reid et al. 2021), a brain region critical for learning and memory processes. However, human research examining the combined effects of PAE and PCE on cognitive development remains limited. Thus, the nature and magnitude of any additive effects remain exploratory. The lack of evidence base for determining whether the effects of co‐use may be uniquely detrimental to human cognitive development leaves public health and policy efforts with little guidance on which prevention strategies may best leverage limited resources in the face of the increasing prevalence of co‐use (e.g., focusing specifically on preventing co‐use rather than on preventing any PAE or PCE).

This study seeks to systematically address each of these limitations by conducting a novel and principled combination of analyses within a large and diverse sample of youth. We use longitudinal growth modeling and three assessments of cognition from a large community‐based sample of adolescents to provide a comprehensive understanding of whether PAE and PCE impact developmental trajectories of cognitive development across adolescence. We test differential and combined effects of PAE and PCE (both binary and frequency of use), include a wide range of covariates in regression models, and perform demographic matching sensitivity analysis to provide a stronger evidence base for whether PAE, PCE, or their combination have adverse effects on cognitive development that are robust to sociodemographic confounders.

### 1.1 Hypotheses

On the basis of prior work showing limited evidence for negative effects of low‐level PAE/PCE on cognitive functioning in the absence of strong demographic controls, we hypothesized that PAE and PCE in this community‐based sample would be generally low and would not be associated with trajectories of adolescent cognition after accounting for sociodemographic covariates. Due to the lack of consensus in the literature regarding the domain‐specific effects of prenatal substance exposure, we did not hypothesize differential effects of PAE/PCE across cognitive abilities. Given limited extant research, analyses examining combined effects of PCE and PAE on adolescent cognition were exploratory.

## 2 Materials and Methods

### 2.1 Procedures and Participants

We analyzed data from ABCD Release 5.1. This release included baseline (*N* = 11,848, *M*_age_ ± SD: 9.92 ± 0.63 years, 48% female), 2‐year follow‐up (*n* = 10,967, *M*_age_ ± SD: 12.03 ± 0.67 years), and 4‐year follow‐up (*n* = 4754, *M*_age_ ± SD: 14.08 ± 0.68 years) assessments. ABCD recruited a probability sample primarily through schools and selected based on sex, race/ethnicity, socioeconomic status, and urbanicity with the aim of recruiting a sample that reflects the diversity of the United States (Garavan et al. 2018). History of severe learning disorder, intellectual disability, autism spectrum disorder (ASD; moderate, severe), pervasive developmental disorder, or other condition requiring repeated or persistent specialized education were exclusionary from ABCD. FASD was not assessed in ABCD and, therefore, was not exclusionary. However, it is possible that individuals with FASD were excluded from participation due to the other exclusion criteria (e.g., due to learning disabilities). Informed parental consent and assent from adolescents were obtained. Procedures were approved by a central Institutional Review Board. This study was not pre‐registered.

### 2.2 Measures

#### 2.2.1 Prenatal Alcohol and Cannabis Exposure

PAE and PCE were assessed using a modified version of the Developmental History Questionnaire (Kessler et al. 2009; Merikangas et al. 2009). Mothers were asked to retrospectively report on their/the child's biological mothers' prenatal alcohol and cannabis use. Questions included: alcohol/cannabis use before and after knowledge of pregnancy (yes/no), maximum number of drinks consumed on a single occasion before and after pregnancy knowledge, average number of drinks consumed per week before and after pregnancy knowledge, and average number of times per day used cannabis before and after pregnancy knowledge. We calculated two PAE variables following methods outlined by Lees et al. (2020) and (Devine et al. 2025): (1) a binary variable reflecting any alcohol use during pregnancy; and (2) an estimate of total number of drinks consumed across pregnancy, which we winsorized at 1.5% to reduce the influence of outliers. Similarly, two PCE variables were calculated: (1) a binary variable reflecting any cannabis use during pregnancy; and (2) an estimate of total number of use occasions across pregnancy, also winsorized at 1.5%.

#### 2.2.2 Cognitive Abilities

A detailed description of the ABCD NIH Toolbox Cognition battery has been reported elsewhere (Luciana et al. 2018). Psychometric studies of these data suggest moderate test–retest reliability for most individual tasks (Anokhin et al. 2022; Taylor et al. 2022), warranting some caution in interpretation of results. The NIH Toolbox consists of seven tasks that assess a variety of cognitive abilities. Due to administration challenges during COVID‐19, only a subset of tests was administered across all assessments. The present analyses utilized measures for which longitudinal assessments were available, including five tests: Flanker Inhibitory Control & Attention Test, Oral Reading Recognition, Pattern Comparison Processing Speed, Picture Sequence Memory, and Picture Vocabulary. Information regarding the cognitive processes assessed by each test is provided in Table 1. We used uncorrected scores because age‐corrected scores would preclude analyses of longitudinal changes.

**TABLE 1:** NIH toolbox tasks and cognitive processes.

Name of test	Cognitive processes assessed
NIH Toolbox Flanker	Cognitive control; attention
NIH Toolbox Oral Reading Recognition Test	Reading ability; language; academic achievement
NIH Toolbox Pattern Comparison Processing Speed	Processing speed; information processing
NIH Toolbox Picture Sequence Memory Test	Visuospatial sequencing and memory
NIH Toolbox Picture Vocabulary Test	Language; verbal intellect

#### 2.2.3 Covariates

We included a wide range of covariates assessed at baseline based on prior evidence of associations with outcomes (Lees et al. 2020). Sociodemographic covariates included adolescent sex (0 = male, 1 = female), adolescent age, adolescent and maternal race (White, Black, Asian, Other/Mixed), adolescent and maternal ethnicity (0 = non‐Hispanic, 1 = Hispanic), highest parental education (1 = < High School, 2 = High School Diploma/GED, 3 = Some College, 4 = Bachelor Degree, and 5 = Post Graduate Degree), and household income (1 = ≤ 50 K, 2 = 50–100 K, 3 = ≥ 100 K). We used dummy‐coded weights to control for the effects of highest parental education, household income, and adolescent and maternal race. Additionally, birthweight, gestational age, maternal medical problems during pregnancy (0 = no, 1 = yes), maternal depression history (0 = no, 1 = yes), parental alcohol problems history (0 = neither parent, 1 = 1 parent, 2 = both parents), and parental drug problems history (0 = neither parent, 1 = 1 parent, 2 = both parents) were included.

We also tested models in which adolescent and maternal race were dichotomized (0 = White, 1 = Non‐White). All results were consistent. We utilized these dichotomized race variables for our matching sensitivity analyses to facilitate generation of the matched subsamples.

### 2.3 Data Analytic Strategy

Descriptive analyses were conducted in R/R Studio (v4.2.3; R Core Team 2023) (Hallquist and Wiley 2018).

#### 2.3.1 Attrition Analyses

To examine whether adolescents who were missing cognitive test data differed on key study variables, we created a binary attrition variable where 0 = complete on all 4‐year follow‐up cognitive data and 1 = missing any 4‐year follow‐up cognitive data, and computed Cohen's *d* values for continuous covariates and odds ratios for binary covariates. Cohen's *d* is a standardized effect size measuring the difference between two group means in standard deviation units, indicating the magnitude of an effect, with guidelines suggesting 0.2 (small), 0.5 (medium), and 0.8 (large) (Cohen 2013).

#### 2.3.2 Growth Models

**FIGURE 1:**
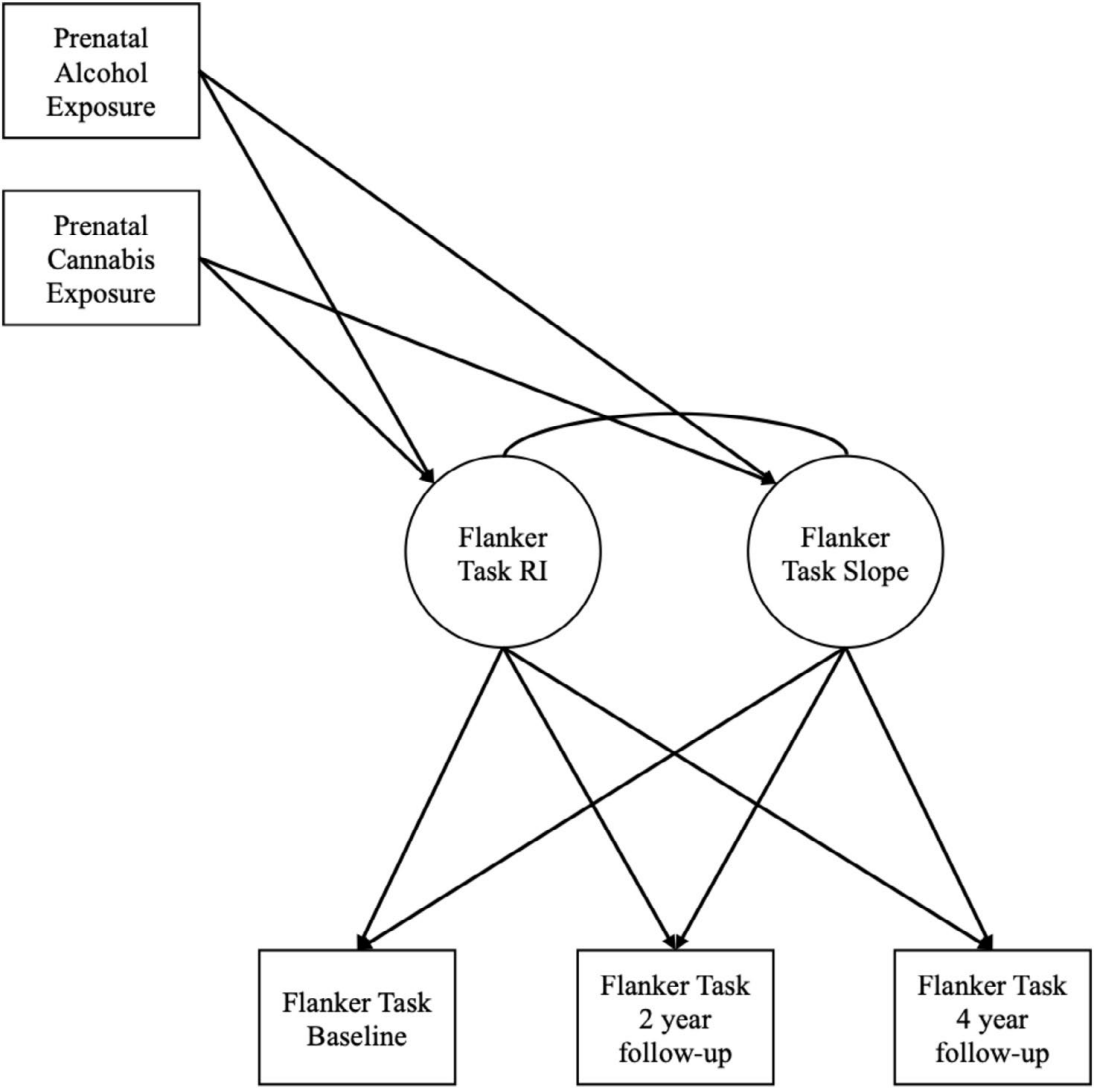
Conceptual figure of prenatal alcohol and cannabis exposure predicting univariate growth curve for flanker task scores. Univariate growth curve for flanker task scores is depicted here; univariate growth curves for all five cognitive abilities were estimated. RI, random intercept.

Structural equation modeling was used to examine growth trajectories for adolescent cognitive abilities and test associations with PAE and PCE (Figure 1).

Model building included several steps. First, univariate growth curves for each cognitive trajectory were estimated. Latent intercepts were specified at baseline, representing uncorrected scores at *M*_age_ = 9.92. We used model fit indices and nested models tests to identify best fitting univariate growth models.

To test main (additive) effects of PAE and PCE, latent growth factors (slopes and intercepts for cognitive ability) were regressed on PAE and PCE variables. Next, we added covariates. This stepwise approach examined whether associations changed after accounting for covariates. Finally, we added a cross‐product term, using centered variables, between PAE and PCE predicting the latent factors to examine dual exposure (multiplicative) effects. Separate models were estimated for each cognitive ability to reduce model complexity. Additionally, we estimated separate models for binary exposure and total use variables to avoid multicollinearity. To correct for multiple tests, we used the Benjamini–Hochberg procedure to calculate *p*‐values adjusted for False Discovery Rate (FDR), with statistical significance set at *p*_fdr_ = < 0.05 (Benjamini and Hochberg 1995). We applied this procedure to five groups of 20 tests each: main effects of binary PAE, binary PCE, total drinks, and total cannabis use, and cross‐product terms.

All structural equation models were specified in Mplus 8.2 using Maximum likelihood with robust standard errors (MLR) to account for non‐normality in PAE and PCE, and a Full‐information Maximum Likelihood estimator (FIML) to account for missing data (Muthén and Muthén 1998). Youths were clustered within families and stratified by study site using the CLUSTER IS and STRATIFICATION IS statements, respectively. Model fit was assessed using Akaike Information Criterion (AIC), sample size‐adjusted Bayesian information Criterion (ssBIC), and chi‐square (smaller values indicate better fit), as well as Comparative Fit Index (CFI), Root Mean Square Error of Approximation (RMSEA), and Standardized Root Mean Square Residual (SRMR). CFI > 0.95, RMSEA ≤ 0.05, and SRMR < 0.06 indicate good model fit (Hu and Bentler 1999).

## 3 Results

### 3.1 Attrition Analyses

Results from attrition analyses are depicted in Table S1. In summary, adolescents from low SES backgrounds were more likely to have missing cognitive data at 4‐year follow‐up. The magnitudes of significant differences were small, however. All main study analyses adjusted for SES characteristics, reducing potential bias due to differential attrition (Graham 2009).

### 3.2 Descriptive Statistics

Descriptive statistics and bivariate correlations are provided in Tables 2 and 3. Adolescents' uncorrected cognitive ability scores were normally distributed and increased across time (Table 2). Alcohol and cannabis use during pregnancy was low. Most mothers reported no prenatal alcohol (*n* = 8257; 74%) or cannabis (*n* = 10,812; 94%) use. Raw data (pre‐winsorization) revealed, on average, women consumed 8.03 drinks and used cannabis 1.42 times throughout their entire pregnancy. Frequency of prenatal alcohol and cannabis use in ABCD was low; the 99th percentiles of prenatal alcohol and cannabis use occasions were 114 and 43 across the full pregnancy, respectively. On average, throughout pregnancy, women who endorsed any prenatal alcohol use reported that they consumed 33.31 drinks and women who endorsed any prenatal cannabis use reported that they used cannabis on 33.00 occasions. Detailed distributions of winsorized total use variables are provided in Figure S1 and Table S14.

**TABLE 2:** Descriptive statistics of cognitive variables.

Variable	*N*	*M*	SD	Min	Max	Skew	Kurtosis
Flanker Task T1	11,715	94	9.15	51	116	−1.00	1.49
Flanker Task T3	8185	100.14	7.71	50	117	−1.00	2.42
Flanker Task T5	3268	104.34	7.31	65	117	−0.93	2.02
Oral Reading T1	11,707	90.86	6.91	59	119	0.00	1.55
Oral Reading T3	10,328	94.97	6.74	67	180	0.41	3.60
Oral Reading T5	4536	99.58	7.14	70	129	0.34	1.06
Pattern Comparison T1	11,697	88.06	14.59	30	140	−0.19	−0.10
Pattern Comparison T3	8146	103.53	15.09	30	163	−0.27	0.48
Pattern Comparison T5	3264	115.66	16.73	35	170	−0.38	0.50
Picture Sequence Memory T1	11,709	102.81	12.07	76	136	0.25	−0.40
Picture Sequence Memory T3	10,398	108.52	12.7	76	136	−0.13	−0.47
Picture Sequence Memory T5	4538	111.55	15.17	74	138	−0.21	−0.71
Picture Vocabulary T1	11,721	84.45	8.12	29	119	0.11	0.64
Picture Vocabulary T3	10,370	88.95	8.52	59	123	0.04	0.16
Picture Vocabulary T5	4566	94.28	8.79	63	126	−0.03	0.02

**TABLE 3:** Bivariate correlation matrix for main study variables.

Variable	1	2	3	4	5	6	7	8	9	10	11	12	13	14	15	16	17	18	19	20	21	22	23	24	25	26	27	28
1. PAE Binary	—																											
2. PCE Binary	0.22	—																										
3. Total Drinks	0.67	0.21	—																									
4. Total CU Occ.	0.10	0.83	0.16	—																								
5. Oral Reading T1	0.10	−0.06	0.06	−0.07	—																							
6. Oral Reading T3	0.09	−0.06	0.05	−0.06	0.76	—																						
7. Oral Reading T5	0.10	−0.06	0.03	−0.07	0.70	0.71	—																					
8. Picture Vocab. T1	0.13	−0.06	0.07	−0.07	0.53	0.53	0.51	—																				
9. Picture Vocab. T3	0.12	−0.07	0.07	−0.08	0.55	0.59	0.56	0.73	—																			
10. Picture Vocab. T5	0.14	−0.06	0.07	−0.08	0.53	0.56	0.58	0.70	0.76	—																		
11. Flanker Task T1	0.06	−0.03	0.04	−0.03	0.28	0.25	0.23	0.26	0.27	0.23	—																	
12. Flanker Task T3	0.07	−0.06	0.04	−0.08	0.28	0.27	0.23	0.27	0.29	0.26	0.44	—																
13. Flanker Task T5	0.07	−0.07	0.03	−0.07	0.29	0.30	0.29	0.28	0.31	0.30	0.35	0.48	—															
14. Pattern Comp. T1	0.01	−0.03	0.01	−0.04	0.20	0.17	0.15	0.19	0.19	0.18	0.37	0.24	0.25	—														
15. Pattern Comp. T3	0.03	−0.03	0.01	−0.02	0.23	0.21	0.15	0.21	0.21	0.18	0.29	0.42	0.28	0.48	—													
16. Pattern Comp. T5	0.04	−0.05	0.00	−0.06	0.24	0.24	0.20	0.22	0.22	0.20	0.29	0.31	0.47	0.41	0.53	—												
17. Picture Seq. T1	0.04	−0.05	0.01	−0.05	0.25	0.22	0.20	0.26	0.26	0.25	0.21	0.18	0.17	0.20	0.21	0.22	—											
18. Picture Seq. T3	0.04	−0.05	0.03	−0.04	0.21	0.22	0.21	0.24	0.27	0.28	0.19	0.20	0.17	0.18	0.23	0.22	0.44	—										
19. Picture Seq. T5	0.04	−0.05	−0.01	−0.05	0.20	0.18	0.20	0.23	0.22	0.27	0.16	0.15	0.21	0.17	0.19	0.23	0.37	0.38	—									
20. Adolescent Sex	0.01	0.01	0.01	0.02	0.00	0.00	0.01	−0.03	−0.03	−0.02	−0.02	−0.03	−0.08	0.06	0.08	0.08	0.06	0.05	0.05	—								
21. Adolescent Age	0.00	−0.02	0.00	−0.02	0.22	0.16	0.12	0.23	0.17	0.14	0.18	0.08	0.10	0.22	0.20	0.21	0.11	0.08	0.07	−0.02	—							
22. Adolescent Race	−0.12	0.10	−0.08	0.10	−0.20	−0.21	−0.18	−0.33	−0.34	−0.32	−0.14	−0.16	−0.17	−0.10	−0.09	−0.12	−0.18	−0.20	−0.15	0.02	−0.01	—						
23. Maternal Race	−0.11	0.10	−0.08	0.10	−0.19	−0.20	−0.17	−0.32	−0.33	−0.31	−0.13	−0.14	−0.17	−0.09	−0.08	−0.13	−0.16	−0.19	−0.13	0.02	−0.01	0.89	—					
24. Ed. < HS	−0.10	0.00	−0.07	0.01	−0.19	−0.18	−0.17	−0.22	−0.23	−0.20	−0.11	−0.12	−0.14	−0.05	−0.05	−0.08	−0.09	−0.10	−0.06	0.02	−0.01	0.15	0.15	—				
25. Ed. Some Coll.	−0.02	0.10	0.02	0.09	−0.14	−0.14	−0.17	−0.17	−0.19	−0.24	−0.07	−0.08	−0.12	−0.07	−0.07	−0.07	−0.10	−0.09	−0.10	−0.01	0.00	0.13	0.13	−0.14	—			
26. Ed. BA	0.03	−0.05	0.03	−0.04	0.08	0.08	0.05	0.11	0.12	0.12	0.07	0.06	0.06	0.04	0.04	0.03	0.05	0.04	0.04	−0.01	0.02	−0.13	−0.13	−0.13	−0.35	—		
27. Ed. Grad.	0.08	−0.10	0.02	−0.10	0.26	0.24	0.25	0.31	0.32	0.32	0.12	0.14	0.16	0.09	0.09	0.12	0.15	0.16	0.13	0.00	0.00	−0.21	−0.21	−0.17	−0.43	−0.42	—	
28. Income Low	−0.13	0.15	−0.06	0.15	−0.29	−0.28	−0.26	−0.36	−0.38	−0.37	−0.18	−0.17	−0.22	−0.11	−0.12	−0.16	−0.17	−0.18	−0.14	0.01	−0.03	0.36	0.37	0.29	0.29	−0.20	−0.40	—
29. Income High	0.12	−0.13	0.06	−0.11	0.24	0.23	0.21	0.31	0.32	0.31	0.15	0.15	0.17	0.09	0.11	0.14	0.16	0.16	0.11	−0.01	0.04	−0.27	−0.26	−0.17	−0.34	−0.34	0.44	−0.55

*Note:* For parsimony, we depict dichotomized adolescent and maternal race (0 = White, 1 = Non‐White) and we do not include correlations with some covariates that were included in hypothesis testing. Parental education dummy variables were coded as less than high school, some college, bachelor's degree, and post graduate degree, with high school diploma or equivalent as the reference group. Household income dummy variables were coded as low income (< $50,000) and high income (≥ $100,000), with middle income ($50,000–$99,999) serving as the reference group.

Abbreviations: BA, bachelor degree; Coll., college; Comp., comparison; CU Occ., cannabis use occasions; Ed., education; Grad., post graduate degree; HS, high school; PAE, prenatal alcohol exposure; PCE, prenatal cannabis exposure; Seq., sequence; Vocab., vocabulary.

Participant characteristics by group (PAE, no PAE, PCE, and no PCE) are provided in Table 4. Adolescents with PAE had parents with higher education, higher household income, and were more likely to be White and non‐Hispanic than adolescents without PAE. Adolescents with PCE had parents with lower education, lower household income, and were more likely to be non‐White than adolescents without PCE.

**TABLE 4:** Prenatal substance exposure characteristics.

Variable	PAE (*N* = 2918; 26%)	No PAE (*N* = 8257; 74%)	PCE (*N* = 693; 6%)	No PCE (*N* = 10,812; 94%)
Highest parental education				
% < HS diploma	1	6	5	5
% HS diploma/GED	6	11	19	9
% Some college	25	27	44	25
% Bachelor degree	28	25	17	26
% Post graduate degree	40	31	15	35
Household income				
% < 50 k	20	34	56	28
% ≥ 50 k and < 100 k	29	28	27	28
% ≥ 100 k	51	38	17	44
Adolescent ethnicity (% Hisp.)	16	22	19	21
Adolescent race				
% Caucasian	84	72	60	85
% Non‐Caucasian	16	28	40	15
Maternal ethnicity (% Hisp.)	12	20	13	18
Maternal race				
% Caucasian	83	71	57	75
% Non‐Caucasian	17	29	43	25
History parental alcohol prob.	*M* = 0.25 (SD = 0.52)	*M* = 0.13 (SD = 0.37)	*M* = 0.46 (SD = 0.67)	*M* = 0.15 (SD = 0.38)
History parental drug prob.	*M* = 0.19 (SD = 0.48)	*M* = 0.10 (SD = 0.34)	*M* = 0.53 (SD = 0.71)	*M* = 0.10 (SD = 0.33)
Medical problems pregnancy	*M* = 0.10 (SD = 0.30)	*M* = 0.08 (SD = 0.27)	*M* = 0.08 (SD = 0.27)	*M* = 0.09 (SD = 0.28)
Birthweight total ounces	*M* = 113.69 (SD = 22.98)	*M* = 111.77 (SD = 23.61)	*M* = 112.07 (SD = 22.98)	*M* = 112.38 (SD = 23.60)
Weeks premature	*M* = 0.79 (SD = 1.93)	*M* = 0.96 (SD = 2.27)	*M* = 0.77 (SD = 1.99)	*M* = 0.93 (SD = 2.20)

*Note:* Parental alcohol/drug problems were coded such that 0 = No parents with history of alcohol/drug problems, 1 = 1 parent with history of alcohol/drug problems, and 2 = Both parents with history of alcohol/drug problems.

Abbreviations: Hisp., hispanic; HS, high school; Prob, problem.

### 3.3 Univariate Growth Models

**FIGURE 2:**
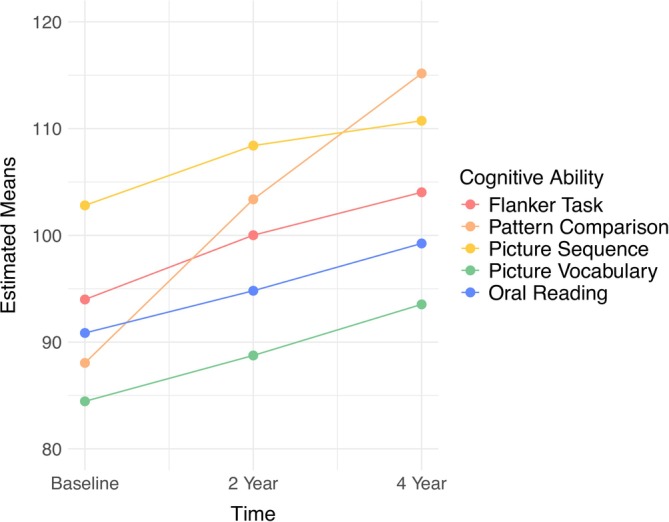
Estimated means of univariate growth curves for cognitive abilities across baseline to 4‐year follow‐up. Uncorrected scores are depicted. Average ages at baseline, 2‐year follow‐up, and 4‐year follow‐up were 9.92, 12.03, and 14.08 years old, respectively.

Estimated means for univariate growth curves are depicted in Figure 2. Examination of sample means for each cognitive ability suggested that they all increased across the three repeated measures (Table 2), and it appeared that the rates of change would be well‐represented by either linear slopes or slopes in which one timepoint was freely estimated (Kline 2016). Compared to linear growth, model fit was superior for models in which growth at the last assessment was freely estimated across all cognitive abilities (Table S2). The univariate growth model for oral reading produced a negative (but nonsignificant) variance for the latent slope factor. After constraining this variance to 0, the model estimated without errors. Nonlinear growth was supported for all cognitive variables (mean slope *p*s all < 0.001). With the exception of the latent slope for oral reading, the variances for the intercept and slope factors of all cognitive variables were statistically significant, indicating individual differences in initial levels and growth in cognition across adolescence. The covariance between the latent random intercept and slope factors was statistically significant for Flanker (*β* = −0.50, *p* < 0.001) and Pattern Comparison (*β* = −0.19, *p* < 0.05) tasks. On average, adolescents who had high initial scores on the Flanker and Pattern Comparison tasks displayed less increases in these scores across time (i.e., regression to the mean).

### 3.4 Prenatal Alcohol Exposure Effects on Cognition

All models testing PAE and PCE effects on adolescent cognition provided good fit (Table S3). Standardized parameter estimates, *p* values from unstandardized model results, and adjusted *p* values are provided in Tables 5 and 6.

**TABLE 5:** Structural equation modeling results for prenatal alcohol and cannabis exposure (binary) predicting cognitive ability scores.

Variable	*β*	SE	*p*	*p* Adj.	*β*	SE	*p*	*p* Adj.	*β*	SE	*p*	*p* Adj.	*β*	SE	*p*	*p* Adj.
	Model 1: Flanker Task Intercept				Model 1: Flanker Task Slope				Model 2: Flanker Task Intercept				Model 2: Flanker Task Slope			
PAE Binary	**0.11**	**0.01**	**< 0.001**	**< 0.001**	−0.01	0.03	0.70	0.78	**0.04**	**0.02**	**< 0.01**	**< 0.05**	−0.02	0.03	0.48	0.64
PCE Binary	**−0.07**	**0.02**	**< 0.001**	**< 0.001**	−0.06	0.04	0.08	0.23	−0.02	0.02	0.67	0.74	−0.02	0.04	0.58	0.68
	**Model 1: Oral Reading Intercept**				**Model 1: Oral Reading Slope**				**Model 2: Oral Reading Intercept**				**Model 2: Oral Reading Slope**			
PAE Binary	**0.14**	**0.01**	**< 0.001**	**< 0.001**	0.47^a^	0.73^a^	0.52	0.65	**0.05**	**0.01**	**< 0.001**	**< 0.001**	−0.04^a^	0.09^a^	0.66	0.78
PCE Binary	**−0.10**	**0.01**	**< 0.001**	**< 0.001**	−0.99^a^	0.21^a^	0.49	0.53	−0.003	0.01	0.84	0.86	−0.08^a^	0.09^a^	0.39	0.49
	**Model 1: Pattern Comp. Intercept**				**Model 1: Pattern Comp Slope**				**Model 2: Pattern Comp. Intercept**				**Model 2: Pattern Comp. Slope**			
PAE Binary	0.03	0.01	< 0.05	0.09	**0.07**	**0.03**	**< 0.01**	**< 0.05**	−0.02	0.02	0.30	0.50	0.04	0.03	0.23	0.46
PCE Binary	**−0.05**	**0.01**	**< 0.001**	**< 0.001**	−0.03	0.03	0.33	0.49	0.004	0.02	0.81	0.85	−0.04	0.03	0.29	0.49
	**Model 1: Picture Seq. Intercept**				**Model 1: Picture Seq. Slope**				**Model 2: Picture Seq. Intercept**				**Model 2: Picture Seq. Slope**			
PAE Binary	**0.08**	**0.02**	**< 0.001**	**< 0.001**	0.05	0.06	0.34	0.52	0.001	0.02	0.96	0.99	0.001	0.07	0.99	0.99
PCE Binary	**−0.09**	**0.01**	**< 0.001**	**< 0.001**	−0.06	0.06	0.36	0.49	0.02	0.02	0.37	0.49	−0.07	0.08	0.38	0.49
	**Model 1: Picture Vocab. Intercept**				**Model 1: Picture Vocab. Slope**				**Model 2: Picture Vocab. Intercept**				**Model 2: Picture Vocab. Slope**			
PAE Binary	**0.18**	**0.01**	**< 0.001**	**< 0.001**	0.03	0.02	0.27	0.49	**0.05**	**0.01**	**< 0.001**	**< 0.001**	−0.03	0.03	0.39	0.56
PCE Binary	**−0.11**	**0.01**	**< 0.001**	**< 0.001**	−0.06	0.03	< 0.05	0.10	0.01	0.01	0.33	0.49	−0.001	0.03	0.99	0.49

*Note:* Model 1 indicates the main effects model for prenatal alcohol and cannabis exposure (binary) predicting cognitive ability scores. Model 2 displays the step‐wise process of adding covariates. For simplicity, regression paths for covariates are not depicted. Covariates included adolescent sex, age, ethnicity, and race, maternal ethnicity and race, highest parental education, household income, weeks born premature, birthweight, medical problems during pregnancy, maternal history of depression, parental history of alcohol use problems, and parental history of drug use problems. Standardized parameter estimates, *p* values from unstandardized model results, and Benjamini‐Hochberg corrected *p* values for False Discovery Rate are depicted. Significant results are bolded.

Abbreviations: Comp., comparison; PAE, prenatal alcohol exposure; PCE, prenatal cannabis exposure; Seq., sequence; Vocab., vocabulary.

^a^ These standardized parameter estimates are likely inflated due to the slope variance of oral reading being fixed to zero.

**TABLE 6:** Structural equation modeling results for prenatal alcohol and cannabis total use occasions predicting cognitive ability scores.

Variable	*β*	SE	*p*	*p* Adj.	*β*	SE	*p*	*p* Adj.	*β*	SE	*p*	*p* Adj.	*β*	SE	*p*	*p* Adj.
	Model 1: Flanker Task Intercept				Model 1: Flanker Task Slope				Model 2: Flanker Task Intercept				Model 2: Flanker Task Slope			
Prenatal Total Drinks	**0.08**	**0.01**	**< 0.001**	**< 0.001**	−0.03	0.03	0.26	0.43	**0.04**	**0.02**	**< 0.01**	**< 0.05**	−0.03	0.03	0.33	0.51
Prenatal Total CU Freq.	**−0.06**	**0.02**	**< 0.001**	**< 0.001**	−0.08	0.04	< 0.05	0.09	−0.02	0.02	0.41	0.68	−0.02	0.04	0.62	0.89
	**Model 1: Oral reading intercept**				**Model 1: Oral reading slope**				**Model 2: Oral reading intercept**				**Model 2: Oral reading slope**			
Prenatal Total Drinks	**0.09**	**0.01**	**< 0.001**	**< 0.001**	−0.82^a^	0.25^a^	0.06	0.13	**0.05**	**0.01**	**< 0.001**	**< 0.001**	**−0.23** ^a^	**0.09** ^a^	**< 0.01**	**< 0.05**
Prenatal Total CU Freq.	**−0.09**	**0.01**	**< 0.001**	**< 0.001**	−0.46^a^	0.38^a^	0.28	0.56	−0.04	0.01	0.05	0.13	−0.04^a^	0.08^a^	0.67	0.89
	**Model 1: Pattern comp. intercept**				**Model 1: Pattern comp slope**				**Model 2: Pattern comp. intercept**				**Model 2: Pattern comp. slope**			
Prenatal Total Drinks	0.03	0.02	0.05	0.13	−0.02	0.03	0.61	0.76	−0.003	0.02	0.88	0.93	−0.02	0.03	0.56	0.75
Prenatal Total CU Freq.	**−0.05**	**0.02**	**< 0.01**	**< 0.01**	−0.01	0.03	0.80	0.94	−0.02	0.02	0.26	0.56	0.03	0.03	0.38	0.68
	**Model 1: Picture seq. intercept**				**Model 1: Picture seq. slope**				**Model 2: Picture seq. intercept**				**Model 2: Picture seq. slope**			
Prenatal Total Drinks	0.03	0.02	0.08	0.16	0.09	0.07	0.19	0.35	0.002	0.02	0.93	0.93	0.03	0.08	0.74	0.82
Prenatal Total CU Freq.	**−0.08**	**0.02**	**< 0.001**	**< 0.001**	−0.03	0.07	0.63	0.89	0.003	0.02	0.88	0.96	−0.02	0.08	0.78	0.94
	**Model 1: Picture vocab. intercept**				**Model 1: Picture vocab. slope**				**Model 2: Picture vocab. intercept**				**Model 2: Picture vocab. slope**			
Prenatal Total Drinks	**0.10**	**0.01**	**< 0.001**	**< 0.001**	0.01	0.02	0.70	0.82	**0.04**	**0.01**	**< 0.01**	**< 0.05**	−0.03	0.03	0.41	0.59
Prenatal Total CU Freq.	**−0.09**	**0.01**	**< 0.001**	**< 0.001**	−0.07	0.03	< 0.05	0.07	0.001	0.01	0.91	0.96	0.001	0.04	0.97	0.97

*Note:* Model 1 indicates the main effects model for prenatal alcohol and cannabis total use occasions predicting cognitive ability scores. Model 2 displays the step‐wise process of adding covariates. For simplicity, regression paths for covariates are not depicted. Covariates included adolescent sex, age, ethnicity, and race, maternal ethnicity and race, highest parental education, household income, weeks born premature, birthweight, medical problems during pregnancy, maternal history of depression, parental history of alcohol use problems, and parental history of drug use problems. Standardized parameter estimates, *p* values from unstandardized model results, and Benjamini‐Hochberg corrected *p* values for False Discovery Rate are depicted. Significant results are bolded.

Abbreviations: Comp., comparison; CU, cannabis use; Freq., frequency; Seq., sequence; Vocab., vocabulary.

^a^ These standardized parameter estimates are likely inflated due to the slope variance of oral reading being fixed to zero.

Before including covariates, binary PAE was positively related to the intercepts for the Flanker, Oral Reading, Picture Sequence, and Picture Vocabulary tasks, such that mothers who reported any prenatal alcohol use had offspring with *higher* baseline scores (*M*_age_ = 9.92). Additionally, binary PAE was related to the Pattern Comparison slope, such that adolescents with PAE had *faster* growth in scores across time. After accounting for covariates, some effects persisted, albeit with smaller effect sizes; PAE was related to higher scores on the intercepts for the Flanker, Oral Reading, and Picture Vocabulary tasks. Several sociodemographic covariates were consistently associated with offspring cognition, and their standardized parameter estimates were small to moderate in size (e.g., parental education *β* range = 0.01–0.36 and household income *β* range = 0.03–0.17; Tables S4–S13).

Results were similar when testing effects of total drinks during pregnancy. Before including covariates, a higher number of prenatal total drinks was positively related to the intercepts for the Flanker, Oral Reading, and Picture Vocabulary tasks. After including covariates, these positive effects were reduced in size but still statistically significant. After including covariates, a negative association between total drinks and the slope for Oral Reading emerged, suggesting mothers who reported a high number of total drinks during pregnancy had offspring who displayed slow growth in Oral Reading across ages 10–14.

#### 3.4.1 Demographic Matching

To further explore the influence of sociodemographic factors on the associations between PAE and adolescent cognitive development, we conducted sensitivity analysis using demographic matching. Due to a lack of representation of certain sociodemographic combinations at the tail ends of cognitive performance in one exposure group but not the other, the assumption of homogeneous regression slopes is violated because the relationship between covariates (i.e., sociodemographic factors) and dependent variables (i.e., cognition) differs across the independent variable (i.e., PAE). Demographic matching mitigates these propensity differences, allowing for more valid estimation of exposure effects and sensitivity analyses. The matching was conducted by first generating a pool of all non‐PAE and non‐PCE participants for each PAE participant who were of the same biological sex, with age difference at baseline no more than 6 months, same binarized race of the child, same highest parental education category, same bracket in household income (if not missing), and same binarized race of the mother (if not missing). Next, a participant from each of those pools was randomly selected. The process was repeated 1000 times and the list with the greatest number of unique participants constituted the control group used in the sensitivity analysis (*n* = 2866). Table 7 depicts the demographic details of the PAE and matched subsamples. Before matching analysis, participants without PAE had parents with lower education (e.g., 31% had a Post Graduate Degree vs. 40% among parents of participants with PAE), had lower household income (e.g., 38% endorsed income above 100 k vs. 51% of participants with PAE), and were less likely to be White (72% vs. 84%) than participants with PAE (Table 4). After matching, there were no differences in highest parental education (e.g., 40% vs. 41% had a Post Graduate Degree), household income (e.g., 51% vs. 49% endorsed income above 100 k), or adolescent binarized race (85% vs. 84% White) across adolescents with and without PAE (Table 7).

**TABLE 7:** Sensitivity analysis using demographic matching: Demographic characteristics.

	PAE (*N* = 2918)		Matched control (*N* = 2866)		Full sample (*N* = 11,848)	
	*N*	%	*N*	%	*N*	%
Biological sex						
Female	1427	48.9%	1396	48.7%	5667	47.8
Male	1491	51.1%	1470	51.3%	6178	52.1
Missing	0	0	0	0	3	0.0%
Age (months)	M = 119	SD = 7.60	M = 119	SD = 7.55	M = 119	SD = 7.50
Education						
< HS diploma	43	1.4%	37	1.3%	592	5.0%
HS diploma/GED	165	5.6%	157	5.5%	1127	9.5%
Some college	714	24.5%	706	24.6%	3068	25.9%
Bachelor	819	28.1%	798	27.8%	3012	25.4%
Post grad degree	1177	40.3%	1168	40.7%	4035	34.1%
Missing	0	0	0	0	14	0.1%
Child bin race						
White	2444	83.8	2427	84.7	8786	74.2%
Non‐White	455	15.6	439	15.3	2888	14.4%
Missing	19	0.6	0	0	174	0.1
Household income						
[< $50 K]	552	18.9%	591	20.6%	3212	27.1%
[≥ $50 K & < $100 K]	797	27.3%	807	28.2%	3066	25.9%
[≥ 100 K]	1419	48.6%	1450	50.6%	4555	38.4%
Missing	150	5.1%	18	0.6%	1015	8.6%
Mother bin race						
White	2008	68.8%	2322	81.0%	7356	62.1%
Non‐White	423	14.5%	455	15.9%	2524	21.3%
Missing	487	16.8%	89	3.1%	1968	16.6%

*Note:* Demographic details of the PAE and matched subsamples, and full sample are depicted.

Abbreviations: HS, high school; *M*, mean; SD, standard deviation.

Next, we re‐ran the structural equation models testing main effects of PAE on offspring cognitive abilities using the PAE and matched data subsets. Consistent with results from the primary models including covariates, binary PAE was significantly positively related to the intercepts for Flanker (*β* = 0.08, *p* < 0.01), Oral Reading (*β* = 0.06, *p* < 0.001), and Picture Vocabulary (*β* = 0.06, *p* < 0.001) tasks, such that mothers who reported any alcohol use during pregnancy had offspring who displayed *higher* scores at baseline (*M*_age_ = 9.92) across these three cognitive domains. Regarding total drinks during pregnancy, results from matching analyses differed somewhat. The positive effect of total drinks during pregnancy on the intercept for Flanker task (*β* = 0.08, *p* < 0.01) persisted. Additionally, a high number of total drinks predicted slower gains in Flanker task scores across time (*β* = −0.08, *p* < 0.05). Given the positive associations of PAE with the intercept of the Flanker task, this slope effect likely represents regression to the mean rather than a detrimental effect of PAE overall. Associations between total drinks on the intercept (*β* = 0.06, *p* < 0.001) and slope (*β* = −0.30, *p* < 0.01) of Oral Reading remained. Likewise, the association between total drinks and the intercept for Picture Vocabulary (*β* = 0.05, *p* < 0.01) persisted using the matched subset of data.

### 3.5 Prenatal Cannabis Exposure Effects on Cognition

Before including covariates, binary PCE was significantly related to the intercepts for the Flanker, Oral Reading, Pattern Comparison, Picture Sequence, and Picture Vocabulary tasks, such that mothers who reported any prenatal cannabis use had offspring who displayed lower scores at baseline (*M*_age_ = 9.92) across all five domains (Tables 5 and 6). Further, binary PCE was related to the Oral Reading slope, such that adolescents with PCE had slower growth in Oral Reading across time. After including covariates, all effects of binary PCE on cognitive abilities were not significant. Here again, several sociodemographic covariates, including parental education and household income, were consistently associated with offspring cognition, and their standardized parameter estimates were small to moderate in size (Tables S4–S13).

Results were similar for total cannabis use occasions. Before including covariates, cannabis use occasions were negatively related to the intercepts for all five cognitive domains. After including covariates, no significant effects remained.

#### 3.5.1 Demographic Matching

We did not perform demographic matching analyses for PCE due to the small sample size of mothers who endorsed PCE (*N* = 693; 6% of sample). Further, fewer PCE effects persisted after including covariates in our regression analyses compared to PAE.

### 3.6 Combined Effects of Prenatal Alcohol and Cannabis Exposure Effects on Cognition

Results for models testing combined PAE and PCE are provided in Tables S4–S13. No cross‐product terms for PAE and PCE (binary or total frequency) were significant in predicting any intercepts or slopes across all five cognitive abilities.

## 4 Discussion

This study utilized a large, longitudinal dataset to examine the differential and combined effects of PAE and PCE on trajectories of cognitive abilities across adolescence, a critical period of cognitive development. This was the first study, to our knowledge, to assess effects of PAE/PCE on developmental trajectories of cognitive development across adolescence, rather than focusing on cross‐sectional cognitive measures. Further, it is the first study to test effects of dual prenatal alcohol and cannabis exposure on adolescent cognition. Additionally, we performed demographic matching analysis to more carefully examine the role of the sociocultural context in which PAE occurs when considering associations with adolescent cognitive outcomes. Binary PAE in the sample was similar (26%) to rates reported in other US samples (18%–27%) (Arria et al. 2006; Meschke et al. 2003, 2008; Piper and Corbett 2012). Binary PCE (6%) was uncommon. Importantly, among women who endorsed any PAE or PCE, use was low‐level (i.e., on average, less than weekly throughout pregnancy). These low levels of PAE and PCE are typical in US community‐based samples (Ko et al. 2015; Shmulewitz and Hasin 2019). Children with especially poor cognition (e.g., severe learning disorder and intellectual disability) were excluded from the ABCD sample. Therefore, results are only generalizable to children within the typical range of cognitive development. Consistent with a large literature (Flak et al. 2014; Shmulewitz and Hasin 2019; Testa 2003), low level PAE was associated with high SES characteristics while low level PCE was associated with low SES characteristics, emphasizing the importance of accounting for the socioeconomic context in which prenatal substance use occurs when examining associations with neurodevelopmental outcomes.

### 4.1 Prenatal Alcohol Exposure Effects

Binary PAE and total number of drinks during pregnancy were positively related to baseline (age 10) cognitive performance. These effects were reduced in size but remained statistically significant after accounting for sociodemographic covariates. On the one hand, these findings are inconsistent with theory and empirical evidence from preclinical and FASD human research that suggests that PAE causes cognitive impairment in offspring through a variety of neurobiological mechanisms (Alhowail 2022; Popova et al. 2023). Notably, animal models of PAE often employ heavy and frequent prenatal exposure to alcohol (i.e., exposure to 95% ethanol vapor [68 mL/h] for 3 consecutive hours every day from GD 5 to GD 20; Reid et al. 2021), reporting mean blood alcohol concentrations as high as 0.17% (Breit et al. 2020). This raises concerns about translating many preclinical findings on the cognitive consequences of PAE, as humans usually report that when they consume alcohol during pregnancy, it is at low levels (Shmulewitz and Hasin 2019).

On the other hand, findings from the current study are consistent with previous human research suggesting that low‐level PAE (e.g., 1–2 drinks/week) has not been reliably linked to adverse cognitive outcomes in offspring (Bandoli 2023; Cheng et al. 2021; Flak et al. 2014; Kelly et al. 2009; Lees et al. 2020). It is implausible that these associations are due to direct, positive neurodevelopmental effects of in utero alcohol exposure. Rather, families with social features positively associated with cognitive ability (e.g., education) may engage in light drinking with few negative cognitive consequences (Shmulewitz and Hasin 2019). Consistent with previous studies in this area (Jacobson et al. 2021), low‐level PAE was associated with high SES characteristics in ABCD. Critically, these SES characteristics—parental education and household income—were positively associated with offspring cognition, and their standardized parameter estimates were generally much larger than those of PAE or PCE. Confounding variables related to these social features (e.g., quality of offspring schooling and nutrition before age 9, parental social support) not captured in this analysis may be contributing to positive associations between low PAE and adolescent cognition. In other words, the number of environmental influences on adolescent cognitive development are vast, and observational studies are unable to measure all possible variables. Therefore, while we included a wide range of sociodemographic covariates in main study models and performed demographic matching sensitivity analyses, the current study was still limited by a failure to capture all factors that are associated with low‐level PAE and high SES, which likely confound associations with adolescent cognitive outcomes.

One negative PAE association emerged. Mothers who reported a high number of total drinks during pregnancy had offspring who displayed slow growth in Oral Reading scores across ages 10–14. However, given that high total drinks were also related to high initial levels of Oral Reading, this may represent regression to the mean.

Importantly, there is evidence of individual differences in genetic susceptibility to alcohol teratogenesis (Dou et al. 2013), and a number of FASD susceptibility genes in both the mother and fetus have been identified (Popova et al. 2023). Therefore, low‐to‐moderate levels of alcohol consumption might be “safe” for one mother and her offspring, but not for her next child or for another maternal‐child pair. In summary, our findings suggest that, on average, low‐level alcohol use during pregnancy is not associated with poor cognitive development across early to middle adolescence; however, previous work investigating individual difference factors that moderate this association suggests that low PAE may still increase risk for poor cognitive development in some cases.

### 4.2 Prenatal Cannabis Exposure Effects

Although there were negative associations between binary PCE and baseline scores across all cognitive abilities and growth in Oral Reading, no effects persisted after including covariates. Here again, these findings are inconsistent with preclinical research indicating that cannabis may impact offspring cognitive development by acting on fetal endocannabinoid systems (Campolongo et al. 2007; Suárez et al. 2004). Yet, these animal models often employ very heavy and frequent doses of THC (e.g., 100 mg/mL at 2 L/min airflow; Breit et al. 2020), which likely do not reflect the typical patterns of PCE documented in humans. Perhaps most importantly, the applicability of preclinical research in this area to humans is considerably limited because such studies cannot account for the complex sociodemographic and cultural factors that shape prenatal substance use. In summary, findings from the current study corroborate recent reviews and meta‐analyses, which conclude the totality of evidence suggests little support for PCE effects on offspring cognitive abilities after accounting for potential confounders (Thompson et al. 2023; Torres et al. 2020).

### 4.3 Combined Effects of Prenatal Alcohol and Cannabis Exposure

No cross‐product terms (binary or total frequency) predicting cognitive abilities were statistically significant. This was not surprising, as no negative PCE main effects persisted after controlling for covariates. Hence, combined low‐level PAE and PCE during pregnancy do not appear to significantly impact youths' cognitive development. Still, this is the first study to investigate the additive and multiplicative effects of PAE and PCE on adolescent cognitive trajectories, and more work is needed to replicate and extend our findings.

### 4.4 Adolescent Cognitive Trajectories

Notably, statistically significant effects of PAE and PCE on adolescent cognition were primarily observed in baseline levels of cognitive ability, rather than in trajectories of cognitive change across time. This pattern of findings is inconsistent with research indicating that substance‐induced deficits may emerge as offspring age and certain brain systems mature (Hagan et al. 2016; Kully‐Martens et al. 2012). Our univariate growth models demonstrated improvement in all five cognitive domains across ages 10–14, supporting well‐accepted developmental theories of adolescence as a critical period characterized by qualitative neurodevelopmental changes (Casey et al. 2008; Steinberg 2008). However, a lack of significant PAE/PCE effects on the growth factors of these cognitive abilities indicates that any influence of PAE and PCE, and the greater sociodemographic context in which prenatal substance use occurs, on offspring cognitive development likely occurs early in childhood and persists in a stable way throughout adolescence. Further, this pattern of results suggests that statistically significant associations between PAE and PCE and adolescent cognition likely pre‐exist the baseline assessment of cognition in ABCD (age 10). Future studies may aim to utilize assessments of cognition that span several developmental periods (e.g., infancy to early adulthood) when investigating the effects of PAE and PCE, and their sociodemographic correlates, on long‐term trajectories of cognition.

## 5 Limitations

First, there are concerns with valid reporting of PAE and PCE. Alcohol and cannabis use during pregnancy is highly stigmatized (English and Greyson 2022; Weber et al. 2021). Underreporting by mothers who indicated no use when they did use may have attenuated associations. Addressing stigma may not simply be overcome through concurrent assessment; some evidence suggests that retrospective reports are more valid than concurrent reports (Alvik et al. 2006). Relatedly, mothers reported on their prenatal cannabis use that occurred between 2006 and 2009. Cannabis potency has increased markedly over the past decade (ElSohly et al. 2021), suggesting low frequencies of modern PCE may produce more pronounced effects that were not observed in this cohort. Second, although children with FASD were not excluded from ABCD, several disorders associated with poor cognition (severe learning disorder, ASD, and intellectual disability) that are highly comorbid with FASD (Popova et al. 2016) were exclusionary. Therefore, there is concern about ceiling effects, as many children at the bottom of the distribution for cognitive abilities were not eligible. Still, our study provides important empirical evidence regarding low‐level PAE and PCE effects on cognition in a large, population‐based, developmentally typical sample of adolescents. Future studies that aim to elucidate the effects of PAE and PCE on long‐term trajectories of adolescent cognition may elect to utilize samples that include mothers who endorse higher levels of prenatal substance use and children who display a wider range of cognitive and developmental deficits. Relatedly, such samples with higher endorsement of prenatal substance use would be better suited to examining more nuanced aspects of exposure (e.g., timing of exposure, stability of exposure throughout pregnancy) on offspring cognitive development. Third, due to administration challenges during COVID‐19, only a subset of NIH toolbox tasks was administered across all longitudinal assessments in ABCD and we were unable to use the standard NIH Toolbox summary measures of general cognitive ability across time. Taken together with evidence of low‐to‐moderate test–retest reliabilities of some individual task data (Anokhin et al. 2022; Taylor et al. 2022), future studies may aim to conduct more parsimonious tests of PAE and PCE effects on cognitive development using metrics of general cognitive ability.

## 6 Conclusions

This was the first study to test differential and combined effects of PAE and PCE on trajectories of adolescent cognitive development and systematically assess the influence of sociodemographic confounds on these associations with the use of matching analyses. This work provides important empirical evidence regarding low‐level PAE and PCE effects on long‐term trajectories of cognition in a large, developmentally typical, population‐based sample of adolescents. Little evidence emerged for negative effects of *low‐level* PCE, PAE, or combined exposure on trajectories of adolescent cognition after accounting for sociodemographic factors. Instead, results suggested small positive associations between low‐level PAE and cognitive abilities, which are likely explained by social factors not measured in this study. Youth from high SES backgrounds likely benefit immensely, with respect to their cognitive development, from environments enriched with good nutrition, high‐quality education, positive experiences with caregivers, early exposure to reading, etc., and these positive influences are likely more influential than any possible negative effects of low‐level PAE. These results, ascertained from analyses using a large, US population‐based sample, emphasize the need for policies and programs aimed at environmental enrichment in disadvantaged communities. Given that adolescence is a sensitive period of brain maturation underlying cognitive abilities, interventions that enrich educational, social, and neighborhood environments may meaningfully support healthy adolescent neurocognitive development. Results suggest light prenatal alcohol and cannabis use are not associated with long‐term negative cognitive outcomes during adolescence and highlight the importance of considering the impact of social factors when studying associations with prenatal substance use.

## Funding

KJP was supported by the National Institute on Alcohol Abuse and Alcoholism (NIAAA; T32AA007477). NER was supported by the National Institute on Drug Abuse (NIDA; T32DA007288). ASW was supported by NIDA K23 DA051561. OK was supported by NIDA K01 DA059598. LM and LMS are supported by funding from NIH's NIAAA (Award numbers: R01AA030575, MPI: Mewton/Squeglia; K24AA031052, PI: Squeglia).

## Disclosure

The authors have nothing to report.

## Conflicts of Interest

The authors declare no conflicts of interest.

## Supporting information


**Figure S1:** Histogram of total drinks during pregnancy winsorised at 1.5% in ABCD sample. *Note*. Winsorised data depicted here. Total number of drinks was winsorised at 1.5% to reduce the influence of outliers. *n* = 146 women fell above the 98.50th percentile and values above this cutoff ranged from 142 to 1260 total drinks across pregnancy.
**Table S1:** Attrition analyses investigating the effect of attrition on key study variables.
**Table S2:** Model fit information for series of univariate growth curves across cognitive domains.
**Table S3:** Model fit information for models testing prenatal alcohol and cannabis exposure on cognitive abilities.
**Table S4:** Structural Equation Modeling results for prenatal alcohol and cannabis exposure (binary) predicting Flanker task scores.
**Table S5:** Structural Equation Modeling results for prenatal alcohol and cannabis exposure (binary) predicting Oral Reading scores.
**Table S6:** Structural Equation Modeling results for prenatal alcohol and cannabis exposure (binary) predicting Pattern Comparison scores.
**Table S7:** Structural Equation Modeling results for prenatal alcohol and cannabis exposure (binary) predicting Picture Sequence scores.
**Table S8:** Structural Equation Modeling results for prenatal alcohol and cannabis exposure (binary) predicting Picture Vocabulary scores.
**Table S9:** Structural Equation Modeling results for prenatal alcohol and cannabis total use occasions predicting Flanker Task scores.
**Table S10:** Structural Equation Modeling results for prenatal alcohol and cannabis total use occasions predicting Oral Reading scores.
**Table S11:** Structural Equation Modeling results for prenatal alcohol and cannabis total use occasions predicting Pattern Comparison scores.
**Table S12:** Structural Equation Modeling results for prenatal alcohol and cannabis total use occasions predicting Picture Sequence scores.
**Table S13:** Structural Equation Modeling results for prenatal alcohol and cannabis total use occasions predicting Picture Vocabulary scores.
**Table S14:** Distribution of total cannabis use occasions during pregnancy winsorised at 1.5% in ABCD sample.

## Data Availability

All data used in the current study from ABCD Study release 5.1 is available to qualified researchers with a completed Data Use Certification on the NIMH Data Archive.
