# Supplementary material for: Associations of Low‐Level Prenatal Alcohol and Cannabis Exposure With Adolescent Cognitive Trajectories
Source: Alcohol Clin Exp Res (Hoboken). 2026 Apr 7;50(4):e70297. doi: 10.1111/acer.70297 (PMC13058172; doi:10.1111/acer.70297)
Supplement: Supplementary file 1 — Figure S1: Histogram of total drinks during pregnancy winsorised at 1.5% in ABCD sample. Note. Winsorised data depicted here. Total number of drinks was winsorised at 1.5% to reduce the influence of outliers. n = 146 women fell above the 98.50th percentile and values above this cutoff ranged from 142 to 1260 total drinks across pregnancy. Table S1: Attrition analyses investigating the effect of attrition on key study variables. Table S2: Model fit information for series of univariate growth curves across cognitive domains. Table S3: Model fit information for models testing prenatal alcohol and cannabis exposure on cognitive abilities. Table S4: Structural Equation Modeling results for prenatal alcohol and cannabis exposure (binary) predicting Flanker task scores. Table S5: Structural Equation Modeling results for prenatal alcohol and cannabis exposure (binary) predicting Oral Reading scores. Table S6: Structural Equation Modeling results for prenatal alcohol and cannabis exposure (binary) predicting Pattern Comparison scores. Table S7: Structural Equation Modeling results for prenatal alcohol and cannabis exposure (binary) predicting Picture Sequence scores. Table S8: Structural Equation Modeling results for prenatal alcohol and cannabis exposure (binary) predicting Picture Vocabulary scores. Table S9: Structural Equation Modeling results for prenatal alcohol and cannabis total use occasions predicting Flanker Task scores. Table S10: Structural Equation Modeling results for prenatal alcohol and cannabis total use occasions predicting Oral Reading scores. Table S11: Structural Equation Modeling results for prenatal alcohol and cannabis total use occasions predicting Pattern Comparison scores. Table S12: Structural Equation Modeling results for prenatal alcohol and cannabis total use occasions predicting Picture Sequence scores. Table S13: Structural Equation Modeling results for prenatal alcohol and cannabis total use occasions predicting Picture Vocabulary scores. T [file ACER-50-0-s001.docx]

| **Supplementary Table 1**  *Attrition analyses investigating the effect of attrition on key study variables.* | | | | | |
| --- | --- | --- | --- | --- | --- |
| ***Variable*** | ***Complete Year 4 Cognitive Data*** | ***Missing Year 4 Cognitive Data*** | ***Cohen's d*** | ***Odds ratio*** | ***p-value*** |
| PAE (Binary) | 1.27 (0.44) | 1.26 (0.44) |  | 0.93 [95% CI = 0.84, 1.02] | 0.12 |
| PCE (Binary) | 1.04 (0.20) | 1.07 (0.25) |  | 1.58 [95% CI = 1.30, 1.92] | <.001 |
| Adolescent Sex | 0.48 (0.50) | 0.48 (0.50) |  | 1.01 [95% CI = 0.93, 1.09] | 0.83 |
| Baseline Age | 119.57 (7.59) | 118.76 (7.45) | -0.11 |  | <.001 |
| Adolescent Race (Binary) | 0.20 (0.40) | 0.27 (0.44) |  | 1.46 [95% CI = 1.32, 1.61] | <.001 |
| Adolescent Ethnicity | 0.21 (0.41) | 0.20 (0.40) |  | 0.98 [95% CI = 0.89, 1.08] | 0.68 |
| Maternal Race (Binary) | 0.22 (0.41) | 0.27 (0.44) |  | 1.35 [95% CI = 1.22, 1.50] | <.001 |
| Maternal Ethnicity | 0.19 (0.39) | 0.17 (0.38) |  | 0.89 [95% CI = 0.79, 0.99] | <.05 |
| Weeks Born Premature | 1.06 (2.35) | 0.87 (2.12) | -0.09 |  | <.001 |
| Maternal Medical Problems During Pregnancy | 0.10 (0.30) | 0.08 (0.27) |  | 0.78 [95% CI = 0.68, 0.90] | <.001 |
| Birthweight (total ounces) | 111.38 (24.45) | 112.63 (23.24) | 0.05 |  | 0.02 |
| Parental Education |  |  |  |  |  |
| % < HS Diploma | 4.3 | 5.3 |  |  |  |
| % HS Diploma/GED | 7.0 | 10.4 |  |  |  |
| % Some College | 25.2 | 26.2 |  |  |  |
| % Bachelor Degree | 27.8 | 24.6 |  |  |  |
| % Post Graduate Degree | 35.7 | 33.5 |  |  |  |
| Household Income |  |  |  |  |  |
| % <50k | 26.7 | 30.8 |  |  |  |
| % ≥50k and <100k | 29.6 | 27.8 |  |  |  |
| % ≥100k | 43.7 | 41.4 |  |  |  |
| Parental Hx. AU Problems |  |  |  |  |  |
| % None | 85.2 | 84.7 |  |  |  |
| % One parent | 13.5 | 13.1 |  |  |  |
| % Both parents | 1.3 | 2.2 |  |  |  |
| Parental Hx. DU Problems |  |  |  |  |  |
| % None | 89.7 | 88.6 |  |  |  |
| % One parent | 8.6 | 9.0 |  |  |  |
| % Both parents | 1.7 | 2.4 |  |  |  |
| *Note*. We created a binary attrition variable where 0=complete on all 4-year follow-up cognitive data and 1=missing any 4-year follow-up cognitive data. Cohen's *d* is a standardized effect size measuring the difference between two group means in standard deviation units, indicating the magnitude of an effect, with guidelines suggesting 0.20 (small), 0.50 (medium), and 0.80 (large). Mean values and (standard deviations) are depicted in columns two and three. Adolescent and maternal ethnicity were coded such that (0=non-Hispanic, 1=Hispanic) and adolescent and maternal race were dichotomized (0=White, 1=Non-White). Parental education dummy variables were coded as less than high school, some college, bachelor’s degree, and graduate degree, with high school diploma or equivalent as the reference group. Household income dummy variables were coded as low income (<$50,000) and high income (≥$100,000), with middle income ($50,000–$99,999) serving as the reference group. PAE=Prenatal alcohol exposure. PCE=Prenatal cannabis exposure. HS=High school. Hx.=History. AU=Alcohol use. DU=Drug use. | | | | | |

| **Supplementary Table 2**  *Model fit information for series of univariate growth curves across cognitive domains.* | | | | | | | |
| --- | --- | --- | --- | --- | --- | --- | --- |
| ***Model*** | ***Model chi-square (df), p-value*** | ***CFI*** | ***RMSEA (95% CI)*** | ***SRMR*** | ***AIC*** | ***ssBIC*** | ***Nested Models Test*** |
| Flanker Task | | | | | | |  |
| Linear | 133.74(3), .00 | 0.93 | 0.06(0.05, 0.07) | 0.11 | 161514.16 | 161539.35 |  |
| Freely estimating time 3 | 17.18(2), .00 | 0.99 | 0.03(0.02, 0.04) | 0.13 | 161378.21 | 161407.60 |  |
| *Nested models test* |  |  |  |  |  |  | 220.07(1), <.001 |
| Oral Reading | | | | | | |  |
| Linear | 11.77(3), .01 | 0.99 | 0.02(0.01, 0.03) | 0.02 | 165558.50 | 165583.71 |  |
| Freely estimating time 3 | 0.89(2), .64 | 1.00 | 0.00(0.00, 0.01) | 0.03 | 165537.06 | 165566.47 |  |
| *Nested models test* |  |  |  |  |  |  | 16.82(1), <.001 |
| Pattern Comparison | | | | | | |  |
| Linear | 112.95(2), .00 | 0.96 | 0.07(0.06, 0.08) | 0.02 | 187690.00 | 187719.38 |  |
| Freely estimating time 3 | 4.675(1), .03 | 1.00 | 0.02(0.00, 0.04) | 0.03 | 187581.43 | 187615.01 |  |
| *Nested models test* |  |  |  |  |  |  | 213.47(1), <.001 |
| Picture Sequence | | | | | | |  |
| Linear | 131.25(2), .00 | 0.96 | 0.07(0.06, 0.08) | 0.04 | 208495.83 | 208525.24 |  |
| Freely estimating time 3 | 0.00(1), .99 | 1.00 | 0.00(0.00, 0.00) | 0.00 | 208367.21 | 208400.82 |  |
| *Nested models test* |  |  |  |  |  |  | 251.31(1), <.001 |
| Picture Vocabulary |  |  |  |  |  |  |  |
| Linear | 17.13(2), .00 | 1.00 | 0.03(0.02, 0.04) | 0.02 | 176858.66 | 176888.07 |  |
| Freely estimating time 3 | 0.37(1), .54 | 1.00 | 0.00(0.00, 0.02) | 0.01 | 176843.04 | 176876.66 |  |
| *Nested models test* |  |  |  |  |  |  | 33.28(1), <.001 |
| *Note*. The univariate growth models for oral reading and flanker task initially produced a negative (but nonsignificant) variance for the latent slope factors. After constraining this variance to 0, the models estimated without errors. | | | | | | | |

| **Supplementary Table 3**  *Model fit information for models testing prenatal alcohol and cannabis exposure on cognitive abilities.* | | | | | | |
| --- | --- | --- | --- | --- | --- | --- |
| ***Model*** | ***Model chi-square (df), p-value*** | ***CFI*** | ***RMSEA (95% CI)*** | ***SRMR*** | ***AIC*** | ***ssBIC*** |
| Flanker Task | | | | | | |
| Binary | 0.86(2), 0.65 | 1.00 | 0.00(0.00, 0.01) | 0.004 | 150730.45 | 150784.16 |
| Binary with covariates | 42.38(24), 0.01 | 0.99 | 0.01(0.00, 0.02) | 0.02 | 102745.54 | 102959.10 |
| Binary PAE x PCE | 42.81(25), 0.01 | 0.99 | 0.01(0.00, 0.02) | 0.02 | 102734.34 | 102955.40 |
| Total use | 0.47(2), 0.79 | 1.00 | 0.00(0.00, 0.01) | 0.01 | 132813.43 | 132865.38 |
| Total use with covariates | 38.43(24), .03 | 0.99 | 0.01(0.00, 0.02) | 0.02 | 96354.70 | 96564.31 |
| Total AU x CU | 40.48(25), .03 | 0.99 | 0.01(0.00, 0.02) | 0.02 | 96356.03 | 96573.00 |
| Oral Reading | | | | | | |
| Binary | 2.25(4), 0.69 | 1.00 | 0.00(0.00, 0.01) | 0.03 | 154735.64 | 154781.12 |
| Binary with covariates | 40.32(26), 0.04 | 1.00 | 0.01(0.00, 0.01) | 0.03 | 103884.22 | 104090.42 |
| Binary PAE x PCE | 40.74(27), 0.04 | 1.00 | 0.01(0.00, 0.01) | 0.03 | 103887.78 | 104101.48 |
| Total use | 3.43(4), 0.49 | 1.00 | 0.00(0.00, 0.01) | 0.03 | 135886.99 | 135930.97 |
| Total use with covariates | 38.36(26), 0.06 | 1.00 | 0.01(0.00, 0.01) | 0.03 | 97450.06 | 97652.42 |
| Total AU x CU | 39.18(27), 0.06 | 1.00 | 0.01(0.00, 0.01) | 0.03 | 97453.19 | 97662.91 |
| Pattern Comparison | | | | | | |
| Binary | 1.04(2), 0.60 | 1.00 | 0.00(0.00, 0.02) | 0.004 | 175410.69 | 175464.38 |
| Binary with covariates | 22.24(24), 0.57 | 1.00 | 0.00(0.00, 0.01) | 0.01 | 120165.36 | 120378.88 |
| Binary PAE x PCE | 24.92(25), 0.47 | 1.00 | 0.01(0.00, 0.01) | 0.01 | 120165.96 | 120386.97 |
| Total use | 3.04(2), 0.22 | 1.00 | 0.01(0.00, 0.02) | 0.01 | 154771.62 | 154823.56 |
| Total use with covariates | 26.98(24), 0.31 | 1.00 | 0.00(0.00, 0.01) | 0.01 | 112733.97 | 112943.54 |
| Total AU x CU | 27.27(25), 0.34 | 1.00 | 0.00(0.00, 0.01) | 0.01 | 112737.15 | 112954.08 |
| Picture Sequence | | | | | | |
| Binary | 2.73(3), 0.26 | 1.00 | 0.01(0.00, 0.02) | 0.01 | 194754.46 | 194808.21 |
| Binary with covariates | 34.07(25), 0.11 | 1.00 | 0.01(0.00, 0.01) | 0.01 | 132571.54 | 132781.48 |
| Binary PAE x PCE | 34.26(26), 0.13 | 1.00 | 0.01(0.00, 0.01) | 0.01 | 132573.66 | 132791.10 |
| Total use | 4.71(3), 0.19 | 1.00 | 0.01(0.00, 0.02) | 0.01 | 171623.31 | 171671.29 |
| Total use with covariates | 31.04(25), 0.19 | 1.00 | 0.01(0.00, 0.01) | 0.01 | 124263.01 | 124469.04 |
| Total AU x CU | 31.53(26), 0.21 | 1.00 | 0.01(0.00, 0.01) | 0.01 | 124266.67 | 124480.06 |
| Picture Vocabulary |  | | | | | |
| Binary | 1.38(3), 0.71 | 1.00 | 0.00(0.00, 0.01) | 0.01 | 165104.06 | 165153.68 |
| Binary with covariates | 23.40(25), 0.55 | 1.00 | 0.00(0.00, 0.01) | 0.01 | 109753.91 | 109963.87 |
| Binary PAE x PCE | 27.23(26), 0.40 | 1.00 | 0.00(0.00, 0.01) | 0.01 | 109757.66 | 109975.11 |
| Total use | 0.09(3), 0.99 | 1.00 | 0.00(0.00, 0.00) | 0.001 | 145525.21 | 145573.19 |
| Total use with covariates | 20.12(25), 0.74 | 1.00 | 0.00(0.00, 0.01) | 0.01 | 102932.28 | 103138.33 |
| Total AU x CU | 21.88(26), 0.70 | 1.00 | 0.00(0.00, 0.01) | 0.01 | 102932.90 | 103146.31 |
| *Note*. The univariate growth models for oral reading initially produced a negative (but nonsignificant) variance for the latent slope factor. After constraining this variance to 0, the models estimated without errors. | | | | | | |

| **Supplementary Table 4**  *Structural Equation Modeling results for prenatal alcohol and cannabis exposure (binary) predicting Flanker task scores* | | | | | | | | | |
| --- | --- | --- | --- | --- | --- | --- | --- | --- | --- |
|  | Model 3: Flanker Task Intercept | | | | | Model 3: Flanker Task Slope | | | |
| *Variable* | *β* | *S.E.* | *p* | *p* Adj. | *β* | | *S.E.* | *p* | *p* Adj. |
| PAE Binary | 0.08 | 0.07 | 0.28 |  | -0.48 | | 0.16 | <.01 |  |
| PCE Binary | -0.001 | 0.02 | 0.95 |  | -0.10 | | 0.05 | 0.05 |  |
| Adolescent sex | -0.01 | 0.02 | 0.55 |  | -0.08 | | 0.03 | <.01 |  |
| Adolescent age | 0.24 | 0.02 | <.001 |  | -0.25 | | 0.04 | <.001 |  |
| Adolescent ethnicity | 0.04 | 0.03 | 0.20 |  | -0.11 | | 0.06 | 0.08 |  |
| Maternal ethnicity | -0.05 | 0.03 | 0.09 |  | 0.09 | | 0.06 | 0.17 |  |
| Adolescent race black | -0.02 | 0.05 | 0.64 |  | -0.12 | | 0.06 | 0.22 |  |
| Adolescent race asian | 0.02 | 0.02 | 0.25 |  | -0.01 | | 0.10 | 0.85 |  |
| Adolescent race other | -0.05 | 0.03 | 0.10 |  | 0.05 | | 0.04 | 0.36 |  |
| Maternal race black | -0.02 | 0.03 | <.01 |  | 0.10 | | 0.06 | 0.34 |  |
| Maternal race asian | 0.02 | 0.05 | 0.04 |  | 0.02 | | 0.10 | 0.71 |  |
| Maternal race other | -0.05 | 0.02 | 0.10 |  | -0.04 | | 0.04 | 0.50 |  |
| Parental educ. < HS | -0.14 | 0.03 | 0.31 |  | -0.03 | | 0.06 | 0.51 |  |
| Parental educ. some coll. | 0.04 | 0.02 | <.01 |  | -0.01 | | 0.05 | 0.87 |  |
| Parental educ. BA | 0.05 | 0.04 | <.001 |  | -0.04 | | 0.07 | 0.55 |  |
| Parental educ. post grad. | -0.02 | 0.04 | <.001 |  | 0.05 | | 0.08 | 0.56 |  |
| Household income low | 0.11 | 0.02 | 0.06 |  | -0.03 | | 0.05 | 0.55 |  |
| Household income high | 0.17 | 0.02 | <.001 |  | -0.01 | | 0.04 | 0.85 |  |
| Weeks premature | -0.04 | 0.02 | 0.07 |  | 0.06 | | 0.04 | 0.18 |  |
| Birthweight | 0.02 | 0.02 | 0.32 |  | 0.04 | | 0.04 | 0.36 |  |
| Medical prob. preg. | -0.01 | 0.02 | 0.67 |  | -0.001 | | 0.03 | 0.97 |  |
| Maternal dep. hx | -0.01 | 0.02 | 0.45 |  | -0.01 | | 0.03 | 0.80 |  |
| Parental AU prob. hx | 0.02 | 0.02 | 0.20 |  | 0.01 | | 0.04 | 0.89 |  |
| Parental DU prob. hx | 0.02 | 0.02 | 0.22 |  | -0.05 | | 0.04 | 0.16 |  |
| PAE x PCE Binary | -0.04 | 0.08 | 0.62 | 0.83 | 0.50 | | 0.17 | <.01 | 0.08 |
| *Note:* Model 3 includes the interaction term for prenatal alcohol and cannabis exposure (binary). Standardized coefficients and *p* values from unstandardized results are presented. Adolescent and maternal race dummy variables were coded as Black, Asian, and Other/Mixed, with White as the reference group. Highest parental education dummy variables were coded as less than high school, some college, bachelor’s degree, and graduate degree, with high school diploma or equivalent as the reference group. Household income dummy variables were coded as low income (<$50,000) and high income (≥$100,000), with middle income ($50,000–$99,999) serving as the reference group. Ethnicity was coded such that 0=Not Hispanic, 1=Hispanic. Birthweight was coded in total ounces. PAE=Prenatal alcohol exposure. PCE=Prenatal cannabis exposure. Prob.=Problems. Preg.=Pregnancy. Hx=History. AU=Alcohol use. DU= Drug use. We used the Benjamini-Hochberg procedure to calculate *p*-values adjusted for False Discovery Rate (FDR) for hypothesis tests; thus, adjusted *p* values are provided in the final column for the interaction term testing the hypothesis that PAE and PCE would have combined effects on cognitive abilities. | | | | | | | | | |

| **Supplementary Table 5**  *Structural Equation Modeling results for prenatal alcohol and cannabis exposure (binary) predicting Oral Reading scores* | | | | | | | | | |
| --- | --- | --- | --- | --- | --- | --- | --- | --- | --- |
|  | Model 3: Oral Reading Intercept | | | | | Model 3: Oral Reading Slope | | | |
| *Variable* | *β* | *S.E.* | *p* | *p* Adj. | *β* | | *S.E.* | *p* | *p* Adj. |
| PAE Binary | 0.07 | 0.05 | 0.15 |  | -0.23 | | 0.37 | 0.53 |  |
| PCE Binary | 0.002 | 0.02 | 0.89 |  | -0.11 | | 0.11 | 0.31 |  |
| Adolescent sex | 0.01 | 0.01 | 0.42 |  | 0.10 | | 0.09 | 0.26 |  |
| Adolescent age | 0.24 | 0.01 | <.001 |  | -0.81 | | 0.05 | <.001 |  |
| Adolescent ethnicity | -0.01 | 0.02 | 0.63 |  | 0.27 | | 0.18 | 0.14 |  |
| Maternal ethnicity | -0.001 | 0.03 | 0.98 |  | 0.02 | | 0.18 | 0.91 |  |
| Adolescent race black | -0.03 | 0.03 | 0.34 |  | -0.26 | | 0.24 | 0.28 |  |
| Adolescent race asian | 0.02 | 0.02 | 0.23 |  | -0.15 | | 0.13 | 0.25 |  |
| Adolescent race other | -0.01 | 0.02 | 0.65 |  | 0.01 | | 0.16 | 0.94 |  |
| Maternal race black | -0.09 | 0.03 | <.05 |  | 0.02 | | 0.25 | 0.93 |  |
| Maternal race asian | 0.08 | 0.02 | <.001 |  | -0.12 | | 0.14 | 0.37 |  |
| Maternal race other | 0.01 | 0.02 | 0.72 |  | -0.07 | | 0.15 | 0.66 |  |
| Parental educ. < HS | -0.07 | 0.02 | <.001 |  | 0.14 | | 0.10 | 0.17 |  |
| Parental educ. some coll. | 0.11 | 0.03 | <.001 |  | 0.14 | | 0.17 | 0.42 |  |
| Parental educ. BA | 0.21 | 0.03 | <.001 |  | 0.35 | | 0.18 | 0.06 |  |
| Parental educ. post grad. | 0.32 | 0.03 | <.001 |  | 0.35 | | 0.20 | 0.08 |  |
| Household income low | -0.08 | 0.02 | <.001 |  | 0.08 | | 0.12 | 0.49 |  |
| Household income high | 0.03 | 0.02 | <.05 |  | -0.02 | | 0.11 | 0.85 |  |
| Weeks premature | -0.02 | 0.02 | 0.21 |  | -0.01 | | 0.10 | 0.94 |  |
| Birthweight | 0.08 | 0.02 | <.001 |  | -0.10 | | 0.11 | 0.35 |  |
| Medical prob. preg. | 0.002 | 0.01 | 0.88 |  | 0.09 | | 0.09 | 0.31 |  |
| Maternal dep. hx | 0.00 | 0.01 | 0.99 |  | 0.07 | | 0.09 | 0.43 |  |
| Parental AU prob. hx | 0.04 | 0.01 | <.01 |  | -0.19 | | 0.09 | 0.05 |  |
| Parental DU prob. hx | -0.03 | 0.02 | 0.10 |  | -0.06 | | 0.10 | 0.56 |  |
| PAE x PCE Binary | -0.03 | 0.05 | 0.57 | 0.83 | 0.21 | | 0.37 | 0.58 | 0.83 |
| *Note:* Model 3 includes the interaction term for prenatal alcohol and cannabis exposure (binary). Standardized coefficients and *p* values from unstandardized results are presented. Adolescent and maternal race dummy variables were coded as Black, Asian, and Other/Mixed, with White as the reference group. Highest parental education dummy variables were coded as less than high school, some college, bachelor’s degree, and graduate degree, with high school diploma or equivalent as the reference group. Household income dummy variables were coded as low income (<$50,000) and high income (≥$100,000), with middle income ($50,000–$99,999) serving as the reference group. Ethnicity was coded such that 0=Not Hispanic, 1=Hispanic. Birthweight was coded in total ounces. PAE=Prenatal alcohol exposure. PCE=Prenatal cannabis exposure. Prob.=Problems. Preg.=Pregnancy. Hx=History. AU=Alcohol use. DU= Drug use. We used the Benjamini-Hochberg procedure to calculate *p*-values adjusted for False Discovery Rate (FDR) for hypothesis tests; thus, adjusted *p* values are provided in the final column for the interaction term testing the hypothesis that PAE and PCE would have combined effects on cognitive abilities. | | | | | | | | | |

| **Supplementary Table 6**  *Structural Equation Modeling results for prenatal alcohol and cannabis exposure (binary) predicting Pattern Comparison scores* | | | | | | | | | |
| --- | --- | --- | --- | --- | --- | --- | --- | --- | --- |
|  | Model 3: Pattern Comparison Intercept | | | | | Model 3: Pattern Comparison Slope | | | |
| *Variable* | *β* | *S.E.* | *p* | *p* Adj. | *β* | | *S.E.* | *p* | *p* Adj. |
| PAE Binary | -0.10 | 0.07 | 0.12 |  | -0.02 | | 0.13 | 0.89 |  |
| PCE Binary | -0.01 | 0.02 | 0.61 |  | -0.05 | | 0.04 | 0.22 |  |
| Adolescent sex | 0.10 | 0.02 | <.001 |  | 0.07 | | 0.03 | 0.03 |  |
| Adolescent age | 0.29 | 0.02 | <.001 |  | -0.02 | | 0.03 | 0.61 |  |
| Adolescent ethnicity | -0.01 | 0.03 | 0.78 |  | -0.004 | | 0.06 | 0.95 |  |
| Maternal ethnicity | -0.01 | 0.03 | 0.79 |  | -0.004 | | 0.06 | 0.95 |  |
| Adolescent race black | -0.04 | 0.05 | 0.37 |  | 0.09 | | 0.10 | 0.39 |  |
| Adolescent race asian | 0.02 | 0.02 | 0.27 |  | 0.02 | | 0.04 | 0.58 |  |
| Adolescent race other | -0.02 | 0.03 | 0.48 |  | 0.01 | | 0.06 | 0.85 |  |
| Maternal race black | -0.08 | 0.05 | 0.09 |  | -0.15 | | 0.10 | 0.14 |  |
| Maternal race asian | 0.03 | 0.02 | 0.10 |  | 0.03 | | 0.04 | 0.43 |  |
| Maternal race other | 0.01 | 0.03 | 0.71 |  | -0.01 | | 0.06 | 0.87 |  |
| Parental educ. < HS | -0.01 | 0.02 | 0.63 |  | 0.02 | | 0.04 | 0.64 |  |
| Parental educ. some coll. | 0.00 | 0.03 | 0.99 |  | 0.14 | | 0.07 | 0.04 |  |
| Parental educ. BA | 0.04 | 0.03 | 0.26 |  | 0.10 | | 0.07 | 0.14 |  |
| Parental educ. post grad. | 0.06 | 0.03 | 0.09 |  | 0.12 | | 0.08 | 0.10 |  |
| Household income low | -0.04 | 0.02 | 0.11 |  | -0.03 | | 0.04 | 0.47 |  |
| Household income high | 0.03 | 0.02 | 0.15 |  | 0.10 | | 0.04 | 0.01 |  |
| Weeks premature | -0.04 | 0.02 | 0.07 |  | 0.003 | | 0.04 | 0.94 |  |
| Birthweight | 0.02 | 0.02 | 0.39 |  | -0.04 | | 0.04 | 0.37 |  |
| Medical prob. preg. | 0.003 | 0.02 | 0.84 |  | -0.02 | | 0.03 | 0.45 |  |
| Maternal dep. hx | -0.02 | 0.02 | 0.21 |  | 0.02 | | 0.03 | 0.44 |  |
| Parental AU prob. hx | 0.01 | 0.02 | 0.79 |  | 0.01 | | 0.03 | 0.67 |  |
| Parental DU prob. hx | -0.01 | 0.02 | 0.45 |  | 0.04 | | 0.04 | 0.20 |  |
| PAE x PCE Binary | 0.09 | 0.07 | 0.18 | 0.75 | 0.06 | | 0.14 | 0.66 | 0.83 |
| *Note:* Model 3 includes the interaction term for prenatal alcohol and cannabis exposure (binary). Standardized coefficients and *p* values from unstandardized results are presented. Adolescent and maternal race dummy variables were coded as Black, Asian, and Other/Mixed, with White as the reference group. Highest parental education dummy variables were coded as less than high school, some college, bachelor’s degree, and graduate degree, with high school diploma or equivalent as the reference group. Household income dummy variables were coded as low income (<$50,000) and high income (≥$100,000), with middle income ($50,000–$99,999) serving as the reference group. Ethnicity was coded such that 0=Not Hispanic, 1=Hispanic. Birthweight was coded in total ounces. PAE=Prenatal alcohol exposure. PCE=Prenatal cannabis exposure. Prob.=Problems. Preg.=Pregnancy. Hx=History. AU=Alcohol use. DU= Drug use. We used the Benjamini-Hochberg procedure to calculate *p*-values adjusted for False Discovery Rate (FDR) for hypothesis tests; thus, adjusted *p* values are provided in the final column for the interaction term testing the hypothesis that PAE and PCE would have combined effects on cognitive abilities. | | | | | | | | | |

| **Supplementary Table 7**  *Structural Equation Modeling results for prenatal alcohol and cannabis exposure (binary) predicting Picture Sequence scores* | | | | | | | | | |
| --- | --- | --- | --- | --- | --- | --- | --- | --- | --- |
|  | Model 3: Picture Sequence Intercept | | | | | Model 3: Picture Sequence Slope | | | |
| *Variable* | *β* | *S.E.* | *p* | *p* Adj. | *β* | | *S.E.* | *p* | *p* Adj. |
| PAE Binary | -0.07 | 0.07 | 0.32 |  | 0.38 | | 0.32 | 0.21 |  |
| PCE Binary | 0.004 | 0.02 | 0.84 |  | -0.001 | | 0.09 | 0.99 |  |
| Adolescent sex | 0.12 | 0.02 | <.001 |  | -0.11 | | 0.08 | 0.12 |  |
| Adolescent age | 0.17 | 0.02 | <.001 |  | -0.17 | | 0.08 | <.05 |  |
| Adolescent ethnicity | -0.04 | 0.03 | 0.24 |  | 0.01 | | 0.14 | 0.97 |  |
| Maternal ethnicity | 0.01 | 0.04 | 0.89 |  | 0.11 | | 0.15 | 0.46 |  |
| Adolescent race black | -0.15 | 0.05 | <.01 |  | -0.04 | | 0.22 | 0.85 |  |
| Adolescent race asian | -0.01 | 0.02 | 0.70 |  | 0.06 | | 0.09 | 0.52 |  |
| Adolescent race other | -0.03 | 0.03 | 0.21 |  | -0.13 | | 0.14 | 0.31 |  |
| Maternal race black | -0.08 | 0.05 | 0.08 |  | -0.24 | | 0.23 | 0.28 |  |
| Maternal race asian | 0.05 | 0.02 | <.05 |  | -0.10 | | 0.09 | 0.23 |  |
| Maternal race other | 0.06 | 0.03 | 0.05 |  | 0.03 | | 0.14 | 0.85 |  |
| Parental educ. < HS | -0.03 | 0.02 | 0.17 |  | -0.03 | | 0.09 | 0.74 |  |
| Parental educ. some coll. | 0.07 | 0.03 | 0.02 |  | -0.01 | | 0.13 | 0.93 |  |
| Parental educ. BA | 0.12 | 0.03 | <.001 |  | 0.12 | | 0.15 | 0.39 |  |
| Parental educ. post grad. | 0.18 | 0.04 | <.001 |  | 0.21 | | 0.17 | 0.19 |  |
| Household income low | -0.05 | 0.02 | <.05 |  | -0.01 | | 0.10 | 0.91 |  |
| Household income high | 0.05 | 0.02 | <.05 |  | 0.00 | | 0.09 | 0.99 |  |
| Weeks premature | -0.03 | 0.02 | 0.21 |  | -0.12 | | 0.09 | 0.14 |  |
| Birthweight | 0.05 | 0.02 | <.05 |  | -0.14 | | 0.10 | 0.13 |  |
| Medical prob. preg. | 0.004 | 0.02 | 0.83 |  | 0.01 | | 0.07 | 0.85 |  |
| Maternal dep. hx | -0.02 | 0.02 | 0.28 |  | 0.07 | | 0.07 | 0.31 |  |
| Parental AU prob. hx | 0.002 | 0.02 | 0.93 |  | -0.10 | | 0.09 | 0.22 |  |
| Parental DU prob. hx | -0.06 | 0.02 | <.001 |  | 0.09 | | 0.09 | 0.32 |  |
| PAE x PCE Binary | 0.08 | 0.07 | 0.30 | 0.75 | -0.41 | | 0.34 | 0.19 | 0.75 |
| *Note:* Model 3 includes the interaction term for prenatal alcohol and cannabis exposure (binary). Standardized coefficients and *p* values from unstandardized results are presented. Adolescent and maternal race dummy variables were coded as Black, Asian, and Other/Mixed, with White as the reference group. Highest parental education dummy variables were coded as less than high school, some college, bachelor’s degree, and graduate degree, with high school diploma or equivalent as the reference group. Household income dummy variables were coded as low income (<$50,000) and high income (≥$100,000), with middle income ($50,000–$99,999) serving as the reference group. Ethnicity was coded such that 0=Not Hispanic, 1=Hispanic. Birthweight was coded in total ounces. PAE=Prenatal alcohol exposure. PCE=Prenatal cannabis exposure. Prob.=Problems. Preg.=Pregnancy. Hx=History. AU=Alcohol use. DU= Drug use. We used the Benjamini-Hochberg procedure to calculate *p*-values adjusted for False Discovery Rate (FDR) for hypothesis tests; thus, adjusted *p* values are provided in the final column for the interaction term testing the hypothesis that PAE and PCE would have combined effects on cognitive abilities. | | | | | | | | | |

| **Supplementary Table 8**  *Structural Equation Modeling results for prenatal alcohol and cannabis exposure (binary) predicting Picture Vocabulary scores* | | | | | | | | | |
| --- | --- | --- | --- | --- | --- | --- | --- | --- | --- |
|  | Model 3: Picture Vocabulary Intercept | | | | | Model 3: Picture Vocabulary Slope | | | |
| *Variable* | *β* | *S.E.* | *p* | *p* Adj. | *β* | | *S.E.* | *p* | *p* Adj. |
| PAE Binary | 0.03 | 0.05 | 0.51 |  | 0.02 | | 0.14 | 0.90 |  |
| PCE Binary | 0.01 | 0.01 | 0.57 |  | 0.01 | | 0.04 | 0.87 |  |
| Adolescent sex | -0.01 | 0.01 | 0.30 |  | 0.02 | | 0.03 | 0.59 |  |
| Adolescent age | 0.27 | 0.01 | <.001 |  | -0.27 | | 0.03 | <.001 |  |
| Adolescent ethnicity | -0.02 | 0.02 | 0.39 |  | -0.03 | | 0.06 | 0.59 |  |
| Maternal ethnicity | -0.08 | 0.02 | <.001 |  | -0.01 | | 0.06 | 0.88 |  |
| Adolescent race black | -0.11 | 0.03 | <.01 |  | -0.20 | | 0.08 | <.01 |  |
| Adolescent race asian | -0.02 | 0.01 | 0.10 |  | 0.03 | | 0.04 | 0.56 |  |
| Adolescent race other | -0.07 | 0.02 | <.001 |  | 0.02 | | 0.05 | 0.72 |  |
| Maternal race black | -0.15 | 0.03 | <.001 |  | 0.06 | | 0.08 | 0.45 |  |
| Maternal race asian | 0.03 | 0.01 | <.05 |  | 0.05 | | 0.04 | 0.21 |  |
| Maternal race other | 0.03 | 0.02 | 0.16 |  | -0.03 | | 0.05 | 0.62 |  |
| Parental educ. < HS | -0.06 | 0.01 | <.001 |  | -0.03 | | 0.04 | 0.49 |  |
| Parental educ. some coll. | 0.12 | 0.02 | <.001 |  | 0.06 | | 0.06 | 0.33 |  |
| Parental educ. BA | 0.23 | 0.02 | <.001 |  | 0.20 | | 0.07 | <.01 |  |
| Parental educ. post grad. | 0.35 | 0.03 | <.001 |  | 0.18 | | 0.07 | 0.01 |  |
| Household income low | -0.08 | 0.02 | <.001 |  | -0.03 | | 0.04 | 0.52 |  |
| Household income high | 0.06 | 0.02 | <.001 |  | -0.05 | | 0.04 | 0.15 |  |
| Weeks premature | -0.03 | 0.02 | 0.05 |  | -0.03 | | 0.04 | 0.40 |  |
| Birthweight | 0.07 | 0.02 | <.001 |  | -0.04 | | 0.04 | 0.34 |  |
| Medical prob. preg. | 0.02 | 0.01 | 0.08 |  | 0.06 | | 0.03 | <.05 |  |
| Maternal dep. hx | 0.03 | 0.01 | <.05 |  | 0.03 | | 0.03 | 0.40 |  |
| Parental AU prob. hx | 0.01 | 0.01 | 0.45 |  | -0.02 | | 0.03 | 0.51 |  |
| Parental DU prob. hx | -0.01 | 0.01 | 0.38 |  | -0.08 | | 0.03 | <.05 |  |
| PAE x PCE Binary | 0.02 | 0.05 | 0.63 | 0.83 | -0.05 | | 0.15 | 0.75 | 0.83 |
| *Note:* Model 3 includes the interaction term for prenatal alcohol and cannabis exposure (binary). Standardized coefficients and *p* values from unstandardized results are presented. Adolescent and maternal race dummy variables were coded as Black, Asian, and Other/Mixed, with White as the reference group. Highest parental education dummy variables were coded as less than high school, some college, bachelor’s degree, and graduate degree, with high school diploma or equivalent as the reference group. Household income dummy variables were coded as low income (<$50,000) and high income (≥$100,000), with middle income ($50,000–$99,999) serving as the reference group. Ethnicity was coded such that 0=Not Hispanic, 1=Hispanic. Birthweight was coded in total ounces. PAE=Prenatal alcohol exposure. PCE=Prenatal cannabis exposure. Prob.=Problems. Preg.=Pregnancy. Hx=History. AU=Alcohol use. DU= Drug use. We used the Benjamini-Hochberg procedure to calculate *p*-values adjusted for False Discovery Rate (FDR) for hypothesis tests; thus, adjusted *p* values are provided in the final column for the interaction term testing the hypothesis that PAE and PCE would have combined effects on cognitive abilities. | | | | | | | | | |

| **Supplementary Table 9**  *Structural Equation Modeling results for prenatal alcohol and cannabis total use occasions predicting Flanker Task scores.* | | | | | | | | | |
| --- | --- | --- | --- | --- | --- | --- | --- | --- | --- |
|  | Model 3: Flanker Task Intercept | | | | | Model 3: Flanker Task Slope | | | |
| *Variable* | *β* | *S.E.* | *p* | *p* Adj. | *β* | | *S.E.* | *p* | *p* Adj. |
| Prenatal Total Drinks | 0.05 | 0.02 | <.01 |  | -0.04 | | 0.03 | 0.17 |  |
| Prenatal Total CU Freq. | -0.01 | 0.02 | 0.71 |  | -0.04 | | 0.04 | 0.30 |  |
| Adolescent sex | -0.01 | 0.02 | 0.61 |  | -0.09 | | 0.03 | <.01 |  |
| Adolescent age | 0.25 | 0.02 | <.001 |  | -0.26 | | 0.04 | <.001 |  |
| Adolescent ethnicity | 0.04 | 0.03 | 0.23 |  | -0.12 | | 0.06 | 0.06 |  |
| Maternal ethnicity | -0.05 | 0.03 | 0.18 |  | 0.10 | | 0.07 | 0.12 |  |
| Adolescent race black | -0.03 | 0.05 | 0.59 |  | -0.14 | | 0.11 | 0.20 |  |
| Adolescent race asian | 0.02 | 0.02 | 0.32 |  | -0.001 | | 0.04 | 0.98 |  |
| Adolescent race other | -0.05 | 0.03 | 0.11 |  | 0.07 | | 0.06 | 0.26 |  |
| Maternal race black | -0.13 | 0.05 | <.05 |  | 0.12 | | 0.11 | 0.28 |  |
| Maternal race asian | 0.04 | 0.02 | <.05 |  | 0.01 | | 0.04 | 0.82 |  |
| Maternal race other | 0.05 | 0.03 | 0.09 |  | -0.06 | | 0.06 | 0.31 |  |
| Parental educ. < HS | -0.02 | 0.02 | 0.33 |  | -0.02 | | 0.05 | 0.64 |  |
| Parental educ. some coll. | 0.10 | 0.04 | <.01 |  | 0.01 | | 0.07 | 0.86 |  |
| Parental educ. BA | 0.16 | 0.04 | <.001 |  | -0.02 | | 0.08 | 0.77 |  |
| Parental educ. post grad. | 0.17 | 0.04 | <.001 |  | 0.06 | | 0.09 | 0.47 |  |
| Household income low | -0.06 | 0.03 | <.05 |  | -0.03 | | 0.05 | 0.48 |  |
| Household income high | 0.07 | 0.02 | <.001 |  | 0.00 | | 0.04 | 0.99 |  |
| Weeks premature | -0.05 | 0.03 | <.05 |  | 0.06 | | 0.04 | 0.19 |  |
| Birthweight | 0.02 | 0.02 | 0.35 |  | 0.03 | | 0.04 | 0.50 |  |
| Medical prob. preg. | -0.01 | 0.02 | 0.58 |  | 0.00 | | 0.03 | 0.99 |  |
| Maternal dep. hx | -0.02 | 0.02 | 0.24 |  | -0.003 | | 0.03 | 0.92 |  |
| Parental AU prob. hx | 0.03 | 0.02 | 0.13 |  | -0.003 | | 0.04 | 0.93 |  |
| Parental DU prob. hx | 0.03 | 0.02 | 0.18 |  | -0.04 | | 0.04 | 0.28 |  |
| Total Drinks x Total CU Freq. | -0.02 | 0.02 | 0.24 | 0.75 | 0.06 | | 0.05 | 0.19 | 0.75 |
| *Note:* Model 3 includes the interaction term for prenatal alcohol and cannabis exposure (binary). Standardized coefficients and *p* values from unstandardized results are presented. Adolescent and maternal race dummy variables were coded as Black, Asian, and Other/Mixed, with White as the reference group. Highest parental education dummy variables were coded as less than high school, some college, bachelor’s degree, and graduate degree, with high school diploma or equivalent as the reference group. Household income dummy variables were coded as low income (<$50,000) and high income (≥$100,000), with middle income ($50,000–$99,999) serving as the reference group. Ethnicity was coded such that 0=Not Hispanic, 1=Hispanic. Birthweight was coded in total ounces. CU Freq.=Cannabis use frequency. Prob.=Problems. Preg.=Pregnancy. Hx=History. AU=Alcohol use. DU= Drug use. We used the Benjamini-Hochberg procedure to calculate *p*-values adjusted for False Discovery Rate (FDR) for hypothesis tests; thus, adjusted *p* values are provided in the final column for the interaction term testing the hypothesis that PAE and PCE would have combined effects on cognitive abilities. | | | | | | | | | |

| **Supplementary Table 10**  *Structural Equation Modeling results for prenatal alcohol and cannabis total use occasions predicting Oral Reading scores.* | | | | | | | | | |
| --- | --- | --- | --- | --- | --- | --- | --- | --- | --- |
|  | Model 3: Oral Reading Intercept | | | | | Model 3: Oral Reading Slope | | | |
| *Variable* | *β* | *S.E.* | *p* | *p* Adj. | *β* | | *S.E.* | *p* | *p* Adj. |
| Prenatal Total Drinks | 0.05 | 0.01 | <.001 |  | -0.24 | | 0.09 | <.01 |  |
| Prenatal Total CU Freq. | -0.02 | 0.01 | 0.16 |  | -0.04 | | 0.09 | 0.65 |  |
| Adolescent sex | 0.01 | 0.01 | 0.28 |  | 0.09 | | 0.09 | 0.29 |  |
| Adolescent age | 0.24 | 0.01 | <.001 |  | -0.79 | | 0.06 | <.001 |  |
| Adolescent ethnicity | -0.01 | 0.02 | 0.56 |  | 0.32 | | 0.19 | 0.09 |  |
| Maternal ethnicity | 0.01 | 0.03 | 0.72 |  | -0.05 | | 0.19 | 0.80 |  |
| Adolescent race black | -0.03 | 0.04 | 0.41 |  | -0.27 | | 0.25 | 0.28 |  |
| Adolescent race asian | 0.02 | 0.02 | 0.26 |  | -0.11 | | 0.13 | 0.42 |  |
| Adolescent race other | -0.01 | 0.02 | 0.53 |  | 0.05 | | 0.16 | 0.74 |  |
| Maternal race black | -0.09 | 0.04 | <.05 |  | 0.02 | | 0.25 | 0.95 |  |
| Maternal race asian | 0.08 | 0.02 | <.001 |  | -0.18 | | 0.13 | 0.19 |  |
| Maternal race other | 0.01 | 0.02 | 0.55 |  | -0.10 | | 0.15 | 0.49 |  |
| Parental educ. < HS | -0.07 | 0.02 | <.001 |  | 0.11 | | 0.11 | 0.29 |  |
| Parental educ. some coll. | 0.12 | 0.03 | <.001 |  | 0.07 | | 0.17 | 0.71 |  |
| Parental educ. BA | 0.22 | 0.03 | <.001 |  | 0.27 | | 0.19 | 0.16 |  |
| Parental educ. post grad. | 0.33 | 0.03 | <.001 |  | 0.24 | | 0.21 | 0.24 |  |
| Household income low | -0.08 | 0.02 | <.001 |  | 0.06 | | 0.12 | 0.63 |  |
| Household income high | 0.03 | 0.02 | <.05 |  | 0.001 | | 0.11 | 0.99 |  |
| Weeks premature | -0.02 | 0.02 | 0.17 |  | -0.02 | | 0.10 | 0.85 |  |
| Birthweight | 0.08 | 0.02 | <.001 |  | -0.11 | | 0.11 | 0.35 |  |
| Medical prob. preg. | -0.01 | 0.01 | 0.74 |  | 0.11 | | 0.09 | 0.23 |  |
| Maternal dep. hx | -0.01 | 0.01 | 0.62 |  | 0.08 | | 0.09 | 0.38 |  |
| Parental AU prob. hx | 0.03 | 0.02 | <.05 |  | -0.15 | | 0.10 | 0.13 |  |
| Parental DU prob. hx | -0.02 | 0.02 | 0.33 |  | -0.08 | | 0.10 | 0.44 |  |
| Total Drinks x Total CU Freq. | -0.01 | 0.02 | 0.39 | 0.83 | 0.02 | | 0.08 | 0.81 | 0.84 |
| *Note:* Model 3 includes the interaction term for prenatal alcohol and cannabis exposure (binary). Standardized coefficients and *p* values from unstandardized results are presented. Adolescent and maternal race dummy variables were coded as Black, Asian, and Other/Mixed, with White as the reference group. Highest parental education dummy variables were coded as less than high school, some college, bachelor’s degree, and graduate degree, with high school diploma or equivalent as the reference group. Household income dummy variables were coded as low income (<$50,000) and high income (≥$100,000), with middle income ($50,000–$99,999) serving as the reference group. Ethnicity was coded such that 0=Not Hispanic, 1=Hispanic. Birthweight was coded in total ounces. CU Freq.=Cannabis use frequency. Prob.=Problems. Preg.=Pregnancy. Hx=History. AU=Alcohol use. DU= Drug use. We used the Benjamini-Hochberg procedure to calculate *p*-values adjusted for False Discovery Rate (FDR) for hypothesis tests; thus, adjusted *p* values are provided in the final column for the interaction term testing the hypothesis that PAE and PCE would have combined effects on cognitive abilities. | | | | | | | | | |

| **Supplementary Table 11**  *Structural Equation Modeling results for prenatal alcohol and cannabis total use occasions predicting Pattern Comparison scores.* | | | | | | | | | |
| --- | --- | --- | --- | --- | --- | --- | --- | --- | --- |
|  | Model 3: Pattern Comparison Intercept | | | | | Model 3: Pattern Comparison Slope | | | |
| *Variable* | *β* | *S.E.* | *p* | *p* Adj. | *β* | | *S.E.* | *p* | *p* Adj. |
| Prenatal Total Drinks | -0.002 | 0.02 | 0.92 |  | -0.02 | | 0.04 | 0.65 |  |
| Prenatal Total CU Freq. | -0.02 | 0.02 | 0.33 |  | 0.04 | | 0.03 | 0.26 |  |
| Adolescent sex | 0.10 | 0.02 | <.001 |  | 0.07 | | 0.03 | 0.03 |  |
| Adolescent age | 0.28 | 0.02 | <.001 |  | -0.03 | | 0.03 | 0.37 |  |
| Adolescent ethnicity | -0.004 | 0.03 | 0.90 |  | -0.02 | | 0.06 | 0.72 |  |
| Maternal ethnicity | -0.004 | 0.03 | 0.89 |  | 0.004 | | 0.06 | 0.95 |  |
| Adolescent race black | -0.05 | 0.05 | 0.32 |  | 0.06 | | 0.10 | 0.56 |  |
| Adolescent race asian | 0.02 | 0.02 | 0.33 |  | 0.04 | | 0.05 | 0.41 |  |
| Adolescent race other | -0.03 | 0.03 | 0.38 |  | 0.01 | | 0.06 | 0.81 |  |
| Maternal race black | -0.07 | 0.05 | 0.15 |  | -0.12 | | 0.11 | 0.25 |  |
| Maternal race asian | 0.03 | 0.02 | 0.12 |  | 0.02 | | 0.04 | 0.65 |  |
| Maternal race other | 0.01 | 0.03 | 0.74 |  | 0.01 | | 0.06 | 0.85 |  |
| Parental educ. < HS | -0.01 | 0.02 | 0.45 |  | 0.04 | | 0.04 | 0.37 |  |
| Parental educ. some coll. | -0.01 | 0.03 | 0.86 |  | 0.17 | | 0.07 | 0.01 |  |
| Parental educ. BA | 0.04 | 0.03 | 0.26 |  | 0.13 | | 0.07 | 0.08 |  |
| Parental educ. post grad. | 0.06 | 0.04 | 0.11 |  | 0.15 | | 0.08 | 0.06 |  |
| Household income low | -0.03 | 0.02 | 0.24 |  | -0.04 | | 0.04 | 0.32 |  |
| Household income high | 0.03 | 0.02 | 0.13 |  | 0.10 | | 0.04 | 0.01 |  |
| Weeks premature | -0.05 | 0.02 | 0.03 |  | -0.004 | | 0.04 | 0.91 |  |
| Birthweight | 0.01 | 0.02 | 0.52 |  | -0.04 | | 0.04 | 0.33 |  |
| Medical prob. preg. | 0.00 | 0.02 | 0.99 |  | -0.01 | | 0.03 | 0.80 |  |
| Maternal dep. hx | -0.03 | 0.02 | 0.07 |  | 0.03 | | 0.03 | 0.34 |  |
| Parental AU prob. hx | 0.01 | 0.02 | 0.67 |  | 0.01 | | 0.04 | 0.69 |  |
| Parental DU prob. hx | -0.01 | 0.02 | 0.60 |  | 0.04 | | 0.04 | 0.24 |  |
| Total Drinks x Total CU Freq. | -0.004 | 0.02 | 0.84 | 0.84 | -0.02 | | 0.03 | 0.47 | 0.83 |
| *Note:* Model 3 includes the interaction term for prenatal alcohol and cannabis exposure (binary). Standardized coefficients and *p* values from unstandardized results are presented. Adolescent and maternal race dummy variables were coded as Black, Asian, and Other/Mixed, with White as the reference group. Highest parental education dummy variables were coded as less than high school, some college, bachelor’s degree, and graduate degree, with high school diploma or equivalent as the reference group. Household income dummy variables were coded as low income (<$50,000) and high income (≥$100,000), with middle income ($50,000–$99,999) serving as the reference group. Ethnicity was coded such that 0=Not Hispanic, 1=Hispanic. Birthweight was coded in total ounces. CU Freq.=Cannabis use frequency. Prob.=Problems. Preg.=Pregnancy. Hx=History. AU=Alcohol use. DU= Drug use. We used the Benjamini-Hochberg procedure to calculate *p*-values adjusted for False Discovery Rate (FDR) for hypothesis tests; thus, adjusted *p* values are provided in the final column for the interaction term testing the hypothesis that PAE and PCE would have combined effects on cognitive abilities. | | | | | | | | | |

| **Supplementary Table 12**  *Structural Equation Modeling results for prenatal alcohol and cannabis total use occasions predicting Picture Sequence scores.* | | | | | | | | | |
| --- | --- | --- | --- | --- | --- | --- | --- | --- | --- |
|  | Model 3: Picture Sequence Intercept | | | | | Model 3: Picture Sequence Slope | | | |
| *Variable* | *β* | *S.E.* | *p* | *p* Adj. | *β* | | *S.E.* | *p* | *p* Adj. |
| Prenatal Total Drinks | 0.00 | 0.02 | 0.99 |  | 0.04 | | 0.09 | 0.67 |  |
| Prenatal Total CU Freq. | 0.00 | 0.02 | 0.99 |  | -0.004 | | 0.08 | 0.96 |  |
| Adolescent sex | 0.11 | 0.02 | <.001 |  | -0.08 | | 0.08 | 0.28 |  |
| Adolescent age | 0.17 | 0.02 | <.001 |  | -0.20 | | 0.10 | <.01 |  |
| Adolescent ethnicity | -0.03 | 0.03 | 0.43 |  | -0.04 | | 0.15 | 0.80 |  |
| Maternal ethnicity | -0.004 | 0.04 | 0.92 |  | 0.15 | | 0.16 | 0.33 |  |
| Adolescent race black | -0.16 | 0.05 | <.01 |  | -0.02 | | 0.23 | 0.93 |  |
| Adolescent race asian | -0.01 | 0.02 | 0.64 |  | 0.08 | | 0.10 | 0.44 |  |
| Adolescent race other | -0.04 | 0.03 | 0.22 |  | -0.12 | | 0.14 | 0.37 |  |
| Maternal race black | -0.07 | 0.05 | 0.16 |  | -0.28 | | 0.25 | 0.24 |  |
| Maternal race asian | 0.05 | 0.02 | <.05 |  | -0.12 | | 0.10 | 0.19 |  |
| Maternal race other | 0.05 | 0.03 | 0.09 |  | 0.02 | | 0.14 | 0.89 |  |
| Parental educ. < HS | -0.03 | 0.02 | 0.17 |  | -0.03 | | 0.09 | 0.75 |  |
| Parental educ. some coll. | 0.06 | 0.03 | 0.05 |  | -0.04 | | 0.14 | 0.80 |  |
| Parental educ. BA | 0.12 | 0.04 | <.01 |  | 0.10 | | 0.16 | 0.51 |  |
| Parental educ. post grad. | 0.17 | 0.04 | <.001 |  | 0.21 | | 0.18 | 0.22 |  |
| Household income low | -0.06 | 0.02 | <.05 |  | -0.02 | | 0.10 | 0.87 |  |
| Household income high | 0.05 | 0.02 | <.05 |  | 0.01 | | 0.10 | 0.89 |  |
| Weeks premature | -0.04 | 0.02 | 0.11 |  | -0.10 | | 0.10 | 0.27 |  |
| Birthweight | 0.04 | 0.02 | 0.05 |  | -0.12 | | 0.10 | 0.22 |  |
| Medical prob. preg. | 0.003 | 0.02 | 0.87 |  | 0.004 | | 0.07 | 0.96 |  |
| Maternal dep. hx | -0.02 | 0.02 | 0.29 |  | 0.05 | | 0.08 | 0.54 |  |
| Parental AU prob. hx | -0.002 | 0.02 | 0.90 |  | -0.07 | | 0.09 | 0.42 |  |
| Parental DU prob. hx | -0.05 | 0.02 | <.01 |  | 0.06 | | 0.09 | 0.54 |  |
| Total Drinks x Total CU Freq. | 0.01 | 0.02 | 0.71 | 0.83 | -0.05 | | 0.10 | 0.62 | 0.83 |
| *Note:* Model 3 includes the interaction term for prenatal alcohol and cannabis exposure (binary). Standardized coefficients and *p* values from unstandardized results are presented. Adolescent and maternal race dummy variables were coded as Black, Asian, and Other/Mixed, with White as the reference group. Highest parental education dummy variables were coded as less than high school, some college, bachelor’s degree, and graduate degree, with high school diploma or equivalent as the reference group. Household income dummy variables were coded as low income (<$50,000) and high income (≥$100,000), with middle income ($50,000–$99,999) serving as the reference group. Ethnicity was coded such that 0=Not Hispanic, 1=Hispanic. Birthweight was coded in total ounces. CU Freq.=Cannabis use frequency. Prob.=Problems. Preg.=Pregnancy. Hx=History. AU=Alcohol use. DU= Drug use. We used the Benjamini-Hochberg procedure to calculate *p*-values adjusted for False Discovery Rate (FDR) for hypothesis tests; thus, adjusted *p* values are provided in the final column for the interaction term testing the hypothesis that PAE and PCE would have combined effects on cognitive abilities. | | | | | | | | | |

| **Supplementary Table 13**  *Structural Equation Modeling results for prenatal alcohol and cannabis total use occasions predicting Picture Vocabulary scores.* | | | | | | | | | |
| --- | --- | --- | --- | --- | --- | --- | --- | --- | --- |
|  | Model 3: Picture Vocabulary Intercept | | | | | Model 3: Picture Vocabulary Slope | | | |
| *Variable* | *β* | *S.E.* | *p* | *p* Adj. | *β* | | *S.E.* | *p* | *p* Adj. |
| Prenatal Total Drinks | 0.04 | 0.01 | <.01 |  | -0.03 | | 0.03 | 0.29 |  |
| Prenatal Total CU Freq. | 0.01 | 0.01 | 0.46 |  | -0.01 | | 0.04 | 0.73 |  |
| Adolescent sex | -0.004 | 0.01 | 0.76 |  | 0.01 | | 0.03 | 0.67 |  |
| Adolescent age | 0.27 | 0.01 | <.001 |  | -0.29 | | 0.04 | <.001 |  |
| Adolescent ethnicity | -0.01 | 0.02 | 0.72 |  | -0.07 | | 0.06 | 0.24 |  |
| Maternal ethnicity | -0.09 | 0.02 | <.001 |  | 0.01 | | 0.06 | 0.90 |  |
| Adolescent race black | -0.11 | 0.03 | <.01 |  | -0.25 | | 0.08 | <.01 |  |
| Adolescent race asian | -0.02 | 0.02 | 0.11 |  | 0.02 | | 0.04 | 0.57 |  |
| Adolescent race other | -0.07 | 0.02 | <.001 |  | -0.003 | | 0.05 | 0.95 |  |
| Maternal race black | -0.15 | 0.03 | <.001 |  | 0.11 | | 0.08 | 0.17 |  |
| Maternal race asian | 0.03 | 0.02 | <.05 |  | 0.06 | | 0.04 | 0.19 |  |
| Maternal race other | 0.03 | 0.02 | 0.10 |  | -0.01 | | 0.05 | 0.92 |  |
| Parental educ. < HS | -0.05 | 0.02 | <.001 |  | -0.02 | | 0.04 | 0.74 |  |
| Parental educ. some coll. | 0.12 | 0.02 | <.001 |  | 0.06 | | 0.07 | 0.39 |  |
| Parental educ. BA | 0.24 | 0.03 | <.001 |  | 0.19 | | 0.07 | <.01 |  |
| Parental educ. post grad. | 0.36 | 0.03 | <.001 |  | 0.17 | | 0.08 | <.05 |  |
| Household income low | -0.08 | 0.02 | <.001 |  | -0.04 | | 0.04 | 0.40 |  |
| Household income high | 0.06 | 0.02 | <.001 |  | -0.06 | | 0.04 | 0.10 |  |
| Weeks premature | -0.03 | 0.02 | <.05 |  | -0.03 | | 0.04 | 0.42 |  |
| Birthweight | 0.07 | 0.02 | <.001 |  | -0.03 | | 0.04 | 0.51 |  |
| Medical prob. preg. | 0.02 | 0.01 | 0.18 |  | 0.06 | | 0.03 | <.05 |  |
| Maternal dep. hx | 0.03 | 0.01 | <.05 |  | 0.01 | | 0.03 | 0.70 |  |
| Parental AU prob. hx | 0.001 | 0.01 | 0.93 |  | -0.02 | | 0.03 | 0.57 |  |
| Parental DU prob. hx | -0.01 | 0.01 | 0.48 |  | -0.07 | | 0.03 | <.05 |  |
| Total Drinks x Total CU Freq. | -0.02 | 0.01 | 0.10 | 0.75 | 0.04 | | 0.04 | 0.29 | 0.75 |
| *Note:* Model 3 includes the interaction term for prenatal alcohol and cannabis exposure (binary). Standardized coefficients and *p* values from unstandardized results are presented. Adolescent and maternal race dummy variables were coded as Black, Asian, and Other/Mixed, with White as the reference group. Highest parental education dummy variables were coded as less than high school, some college, bachelor’s degree, and graduate degree, with high school diploma or equivalent as the reference group. Household income dummy variables were coded as low income (<$50,000) and high income (≥$100,000), with middle income ($50,000–$99,999) serving as the reference group. Ethnicity was coded such that 0=Not Hispanic, 1=Hispanic. Birthweight was coded in total ounces. CU Freq.=Cannabis use frequency. Prob.=Problems. Preg.=Pregnancy. Hx=History. AU=Alcohol use. DU= Drug use. We used the Benjamini-Hochberg procedure to calculate *p*-values adjusted for False Discovery Rate (FDR) for hypothesis tests; thus, adjusted *p* values are provided in the final column for the interaction term testing the hypothesis that PAE and PCE would have combined effects on cognitive abilities. | | | | | | | | | |

Supplementary Figure 1. Histogram of total drinks during pregnancy winsorised at 1.5% in ABCD sample.


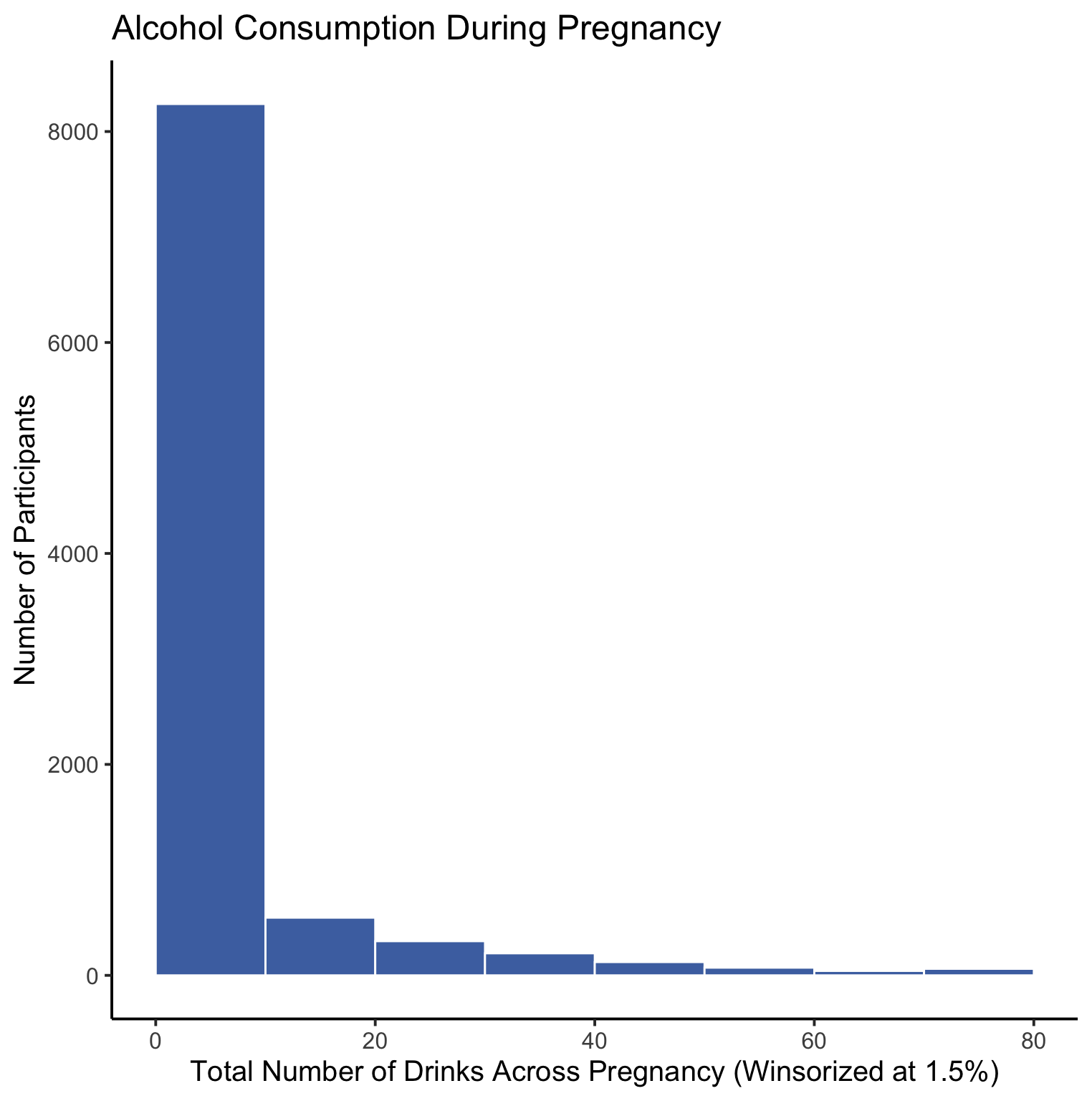


*Note*. Winsorised data depicted here. Total number of drinks was winsorised at 1.5% to reduce the influence of outliers. *n*=146 women fell above the 98.50^th^ percentile and values above this cutoff ranged from 142 to 1,260 total drinks across pregnancy.

| **Supplementary Table 14**  *Distribution of total cannabis use occasions during pregnancy winsorised at 1.5% in ABCD sample.* | | |
| --- | --- | --- |
| ***Number of Cannabis Use Occasions*** | ***N endorsed*** | ***Percent of ABCD endorsed*** |
| 0 (No use) | 9803 | 82.6 |
| 0.1–1 | 10 | 0.08 |
| 1.1–5 | 67 | 0.56 |
| 5.1–10 | 85 | 0.72 |
| 10.1–20 | 80 | 0.67 |
| 20.1–24 | 169 | 1.42 |
| *Note*. Winsorised data depicted here. Total cannabis use occasions was winsorised at 1.5% to reduce the influence of outliers. n=153 women fell above the 98.50th percentile and values above this cutoff ranged from 25 to 308 total cannabis use occasions across pregnancy. Values were grouped into meaningful categories for descriptive purposes. Counts (n) and percentages (%) reflect the number and proportion of participants in each category. | | |
